# From forest to farm: the impact of a broad spectrum of lifestyles on the porcine gut microbiota

**DOI:** 10.1016/j.crmicr.2026.100576

**Published:** 2026-02-28

**Authors:** Luke Comer, Peiyang Huo, Camillo Colleluori, Haoran Zhao, Muhammad Zeeshan Akram, Romario Florent Kpossou, Ester Arévalo Sureda, Jan Aerts, Nadia Everaert

**Affiliations:** a Nutrition & Animal Microbiota EcoSystems Lab, Department of Biosystems, KU Leuven, 3001 Heverlee, Belgium; b Augmented Intelligence for Data Analytics Lab, Department of Biosystems, KU Leuven, 3001 Heverlee, Belgium; c Laboratory of the Regional Centre of Excellence in Poultry Science, Department of Animal Science and Veterinary, University of Lomé, Lomé, Togo

**Keywords:** Gut microbiota, Porcine microbiota, Pigs, Husbandry, Enterotype

## Abstract

The gut microbiota is shaped profoundly by the diet, environment, and genetics of its host. Yet, previous studies have focused primarily on investigating factors individually in standardised facilities. In the present study, faecal samples and rearing metadata were collected from a highly-diverse cohort of 344 pigs, from farms to the wild, spanning eight countries and two continents. We hypothesised that varied lifestyles would induce a range of diverse microbiotas. Rustic rearing conditions were associated with increased α-diversity, alongside *Treponema*, while conventionally-reared pigs exhibited increased *Lactobacillus*. Equally, both rustic-associated bacteria and conventional feed were associated with increased short-chain fatty acids. Gas production kinetics from *in vitro* fermentation revealed the complex role of underexplored bacteria in fibre fermentation. The present study represents one of the most diverse single-study cohorts of pigs hitherto, and underscores the complex interplay between swine husbandry and the gut microbiota, and in turn, its potential consequences for functionality.

## Highlights

• Dietary variables had the largest effect on microbiota composition.
• Rustic and conventional rearing conditions led to differential microbiota profiles.
• Rustic rearing favoured *Treponema*-enriched, more-diverse microbial profiles.
• Enterotypes were associated with husbandry conditions and faecal water content.
• Underexplored taxa contributed to *in vitro* fibre fermentation and SCFA production.

## Graphical abstract

**Figure:**
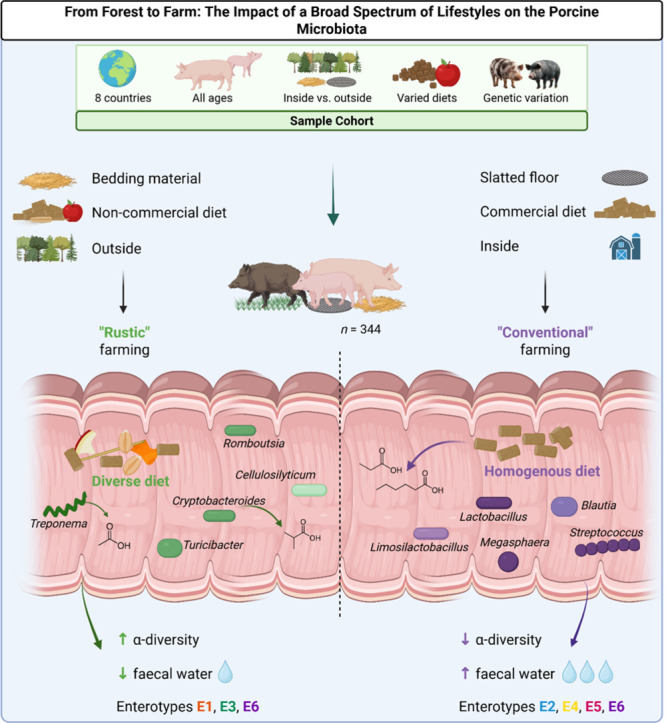

**Abbreviations**
**A**: Maximum gas volume (gas kinetics)
**ASV**: Amplicon sequence variant
**B**: Inflection parameter (gas kinetics)
**BCFA**: Branched-chain fatty acid
**C**: Rate constant of gas production (gas kinetics)
**E (1-6)**: Enterotype (enterotypes 1-6)
**LEfSe**: Linear discriminant analysis effect size
**MaAsLin2**: Microbiome multivariable association with linear models 2
**MC**: Microbial component
**MCFA**: Medium-chain fatty acid
**PERMANOVA**: Permutational multivariate analysis of variance
**PCoA**: Principal coordinates analysis
**R_MAX_**: The maximum rate of gas production (gas kinetics)
**SCFA**: Short-chain fatty acid
**T_MAX_**: Time to reach the maximum rate of gas production (gas kinetics)
**VFA**: Volatile fatty acid

## 1 Background

The gut microbiota occupies a precarious and ever-changing interface, dependent on both the external world and its host for survival. Understanding how varied lifestyles, in the form of diet and environment shape the microbiota is vital, given the microbiota’s ultimate impact on the health of its host. Studies analysing animals with starkly contrasting lifestyles have demonstrated the significant impact of lifestyle on microbiota composition. Comparisons of farmed pigs for instance with feral pigs, wild boar, and even warthogs have shown significant, large lifestyle-induced differences (Correa-Fiz et al., 2019; Fenske et al., 2020; Petrelli et al., 2022; Ushida et al., 2016; Wei et al., 2022). Yet, while revealing, these studies do not address which aspect of the lifestyle specifically leads to the particular changes observed.

On the contrary, numerous individual factors have been assessed across a multitude of separate studies. Diet, particularly crude fibre and protein content, has been well studied for its impacts on the microbiota, often in standardised experimental settings (Patil et al., 2020; Pollock et al., 2021; Tang et al., 2024). Breed, sex, weaning age, the provision of bedding material, and soil exposure have all been analysed for their microbiota-influencing properties (Holman et al., 2021; Patil et al., 2020; Vo et al., 2017; Wen et al., 2021). Nevertheless, taken alone, these studies can have contradictory findings. Furthermore, by addressing very specific factors, these studies often fail to account for other microbial differences arising from interstudy lifestyle variation. Early studies for instance found outdoor-born pigs to have greater microbial diversity post-weaning, a trend verified more recently, albeit with the two groups comprising different breeds (Holman et al., 2023; Pluske et al., 2007). In contrast, no difference was found between indoor- and outdoor-reared Krškopolje pigs, instead finding farm of origin a stronger influence on the microbiota (Papić et al., 2025). Equally, the provision of bedding material was found to have contradictory results, in some cases driving taxonomic differences, while other times proving insignificant (Kubasova et al., 2017; Megahed et al., 2019; Wen et al., 2021).

A limited number of studies have indeed attempted to bridge the gap hitherto, assessing pigs across a range of farming systems and analysing the impact of multiple lifestyle factors. Wang *et al.* for instance assessed pigs across three farms and found diet to be the most significant influence of the microbiota, above age, interindividual variation, and sex (Wang et al., 2019). Kuthyar *et al.* also assessed pigs and their wild relatives across a range of environments, and demonstrated domestication context as a key driver of α-diversity, more influential than outside access or antibiotic use (Kuthyar et al., 2023). Further specific day-to-day husbandry variables however remain under-investigated.

Moving beyond taxonomic identification and better understanding the functionality of the microbiota for its host remains crucial. While microbiota-produced metabolites such as short-chain fatty acids (SCFAs) have been thoroughly studied in pigs (Sebastià et al., 2024), other parameters such as faecal water content, which have gained traction in the human field as an important influence (Falony et al., 2016; Lokmer et al., 2020; Procházková et al., 2023), remain relatively unexplored in pigs outside of disease contexts.

The present study sought to investigate the microbiota across a spectrum of real-world conditions, analysing which factors influence the microbiota, and understanding potential impacts on the host. We hypothesised that the dietary and environmental factors would produce both independent and synergistic effects on the porcine microbiota, reflected in altered composition and potential functionality in terms of SCFA production.

## 2 Materials and methods

### 2.1 Sample collection

Faecal samples were collected from pigs as a field study. As such, animals were not controlled per group of each variable measured, but were chosen from a spectrum of different lifestyles and conditions. In total, samples were obtained from a cohort of 344 pigs (*Sus scrofa domesticus*) and other members of the family Suidae, namely Visayan warty pigs (*Sus cebifrons, n =*4) and North Sulawesi babirusas (*Babyrousa celebensis n =*4). Samples were collected from each animal just once.

Fresh faecal samples were collected either upon defaecation naturally, or via rectal stimulation. Any faeces which came into contact with the external environment was discarded. Immediately afterwards, the faeces was snap-frozen in liquid nitrogen and kept at −80 °C. Animals were unharmed, and anaesthesia/euthanasia were not performed.

At each location, the farmer/keeper was consulted and a questionnaire was completed to gather information about key metadata variables including the animals’ age, breed, sex, environment, diet, and health (appendix A). Given a lack of standardisation between countries and farming systems, age categories were defined according to the ages shown in Table S1. Animals were also grouped into dietary categories according to crude fibre and protein content in the feed, when this was known to the farmers. Dietary classifications were made according to the criteria in Table S2.

Alongside farmed and captive animals, the final cohort also consisted of 15 wild boar (*Sus scrofa*) shot in Heverleebos and Meerdaalwoud, Oud-Heverlee, Belgium in association with the Instituut voor Natuur- en Bosonderzoek. Following the hunt, animals were eviscerated, and the intestines were promptly transported to the laboratory on ice whereupon the rectal contents were obtained and snap-frozen in liquid nitrogen prior to storage at −80°C.

### 2.2 Samples transported in nucleic acid preservation buffer

For 30 of the 344 animals where immediate snap-freezing was not possible, samples were transported in a nucleic acid preservation buffer. The buffer was made following a previous protocol (Camacho-Sanchez et al., 2013). In short, a solution was prepared consisting of 0.74% EDTA disodium salt dihydrate (C_10_H_14_N_2_Na_2_O_8_·2H_2_O), 0.74% sodium citrate trisodium salt dihydrate (C_6_H_5_Na_3_O_7_·2H_2_O), and 70% ammonium sulphate ((NH_4_)_2_SO_4_) in ultrapure, distilled water. The pH was adjusted to 5.2 with sulphuric acid. Solution was prepared and decanted into sterile containers shortly before use.

Following defaecation, the sample was placed in the tube of buffer and kept at room temperature for no more than 10 days. Once at the lab, the samples were centrifuged in sterile phosphate-buffered saline at 3000 × g for 10 min, drained, aliquoted and stored at −80°C. A pilot comparison study (*n =*8 sows) was performed (unpublished) prior to this which confirmed negligible differences in microbiota composition as a result of this method of storage, in accordance with previous literature (Menke et al., 2017). These buffer-transported samples were however not analysed for volatile fatty acids (VFAs) or faecal water content.

### 2.3 DNA extraction

DNA was extracted from 0.4 g faeces using the QIAamp PowerFecal Pro Plus kit (Qiagen Benelux, Venlo, Netherlands), with all other steps followed in accordance with the manufacturer’s instructions. Following extraction, the concentration and purity of the DNA was measured using a spectrophotometer (SimpliNano (Biochrom), Massachusetts, United States) and 1% agarose gel electrophoresis was performed to assess structural integrity. DNA was stored at −20°C hereafter.

### 2.4 16S rRNA gene amplicon sequencing

16S rRNA gene amplicon sequencing of the V1-V9 regions was performed by the VIB Nucleomics Core (Leuven, Belgium) using the Pacific Biosciences (PacBio) platform. The primers 27F: AGRGTTYGATYMTGGCTCAG and 1492R: RGYTACCTTGTTACGACTT were used, and a sample-specific combination of barcodes was also applied. A ZymoBIOMICS® microbial community standard was included as a positive control. Negative controls from both the DNA extraction process and the sequencing facility were also included. Sequencing was performed in four separate runs over time.

### 2.5 Volatile fatty acid analysis

VFAs were extracted from approximately 350 mg faeces, as described previously (Comer et al., 2025), with the inclusion of a 2-methylhexanoic internal standard was used. Standards were prepared using a stock solution (Supelco’s Volatile Free Acid Mix) containing acetate, propionate, isobutyrate, butyrate, isovalerate, valerate, isocaproate, caproate and enanthate.

The VFA composition was then determined by gas chromatography on an HP 6890 Series GC System (for digesta and faecal samples) and a Shimadzu GC-2050 (for fermentation samples) using nitrogen as a carrier gas. All concentrations were corrected for the exact mass of the sample and expressed in mM/g. Total SCFAs were defined as the sum of acetate, propionate, butyrate, and valerate. Total branched-chain fatty acids (BCFAs) comprised isobutyrate, isovalerate, and isocaproate. Total medium-chain fatty acids (MCFAs) consisted of caproate and enanthate, while total VFAs comprised all VFAs (acetate to enanthate, including BCFAs).

### 2.6 Water content determination

Water content was determined as described previously (Comer et al., 2025), using 300 mg sample, and lyophilising for 48 hours (Christ Alpha 1-4 machine, Osterode am Harz, Germany). In addition to the numerical value of water content, water content categories were established as a porcine equivalent of the Bristol stool score scale commonly used in human research. Categories were calculated by plotting the total range of water content values, and categorising ± 5%, 10%, and 20% deviation from the median (Figure S1). This produced six porcine stool scores: from score 1 consisting of hard, dropping-like stools (< 59.22% water content) to score 6 consisting of the wettest samples (> 81.43% water content).

### 2.7 Microbiota analysis

Sequencing data were obtained in fastq.gz file format. Following initial inspection via the FASTQC programme, sequencing data were processed using the Nextflow pipeline as recommended by PacBio for HiFi full-length sequencing data, using QIIME2 with DADA2. The Nextflow HiFi-16S-workflow pipeline (https://github.com/PacificBiosciences/HiFi-16S-workflow v. 07) was run using the high performance computing platform provided by the VSC (Flemish Supercomputer Centre). Taxonomic classification was performed using a naïve Bayes classifier and a flexible VSEARCH taxonomic database combining SILVA (v. 138.1), GTDB (r207), and RefSeq + RDP (v. 16).

Subsequent analysis was performed in RStudio using R (v. 4.4.3). Amplicon sequence variants (ASVs) found in the negative controls were identified as contaminants and removed from all samples. This included for example ASVs belonging to the species *Ralstonia mannitolilytica, Acidovorax* sp.*00032535,* and *Bacteroides ndongoniae.* Sequencing depth was deemed significant in all samples according to rarefaction curves plotting α-diversity (Figure S2). Diversity area modelling was plotted as the number of observed genera against the number of samples. The core microbiota was calculated as the taxa shared by > 95% individuals. α-diversity was calculated according to the Chao1, Pielou evenness, Simpson, and Shannon indices (phyloseq R package). Bray-Curtis distance was used to calculate β-diversity which was plotted as a principle coordinate analysis (PCoA).

Linear discriminant analysis effect size (LEfSe) was performed using the R package microbiomeMarker to determine significant associations of genera with particular variables, with an LDA cutoff of ≥ 2.5 indicating significance. For multivariate testing, microbiome multivariable association with linear models (MaAsLin2) analysis was performed using the Maaslin2 package.

PICRUSt2 was performed after removing low-count reads (read counts ≤ 5 in all samples) to obtain predicted metagenomic microbiome functions using the online Galaxy platform, made available by Deng *et al*. (https://dmap.denglab.org.cn/).

### 2.8 Lifestyle and metadata analysis

Permutational multivariate analysis of variance (PERMANOVA) with 9999 permutations was used to determine the significance of associations of lifestyle factors with β-diversity, and the PERMANOVA ω^2^ effect size was calculated for categorical variables using the R package MicEco. Random forest classification (categorical variables) and regression (numerical variables) was performed using the randomforest R package, (set.seed= 42). Multiple correspondence analysis was performed on categorical variables using the FactoMineR package. The φ test was used to determine the strength of association between two categorical variables, and Cramér’s V test for three or more categorical variables. For determining the significance of associations between such categorical variables, the χ^2^ test was performed.

Correlations between the microbiota, VFAs and dietary factors were performed via the Spearman rank test. Selected correlations were then visualised via a chord diagram, plotted using the circlize package in R.

### 2.9 Batch effect correction

Considering the inclusion of the samples in four separate sequencing runs, efforts were made to reduce any potential batch effects of sequencing run, using age category as a correcting factor. Eight batch effect correction algorithms were tested following centred-log ratio normalisation: BMC, ComBat, ConQuR, MMUPHin, PLSDA-batch, sPLSDA-batch, rBE, and SVD. The different methods were compared, following adapted methodology of Wang and Lê Cao, considering silhouette coefficients, alignment scores, variance, redundancy analysis and principal variance component analysis (https://github.com/EvaYiwenWang/PLSDAbatch) (Wang and Lê Cao, 2023). Wang and Lê Cao’s sPLSDA-batch was chosen as the most appropriate method, resulting in a modest reduction in ω^2^ effect size of sequencing run from 6.77% to 5.52%. This remaining effect was not considered spurious given that there was partial genuine confounding between biology and methodology, with pigs from the same sampling location generally more likely to be included in the same sequencing batch. As such, caution was taken to avoid over-correction as highlighted previously (Hui et al., 2024). Batch-effect-corrected data were used exclusively for comparative multivariate analysis where relative differences between samples were deemed essential to the data’s overall structure: PCoA of β-diversity, and lifestyle factor analysis (ω^2^ effect size, and random forest classification). In contrast, the original, uncorrected data were used for all other univariate and composition-based analysis such as taxonomy and α-diversity to avoid the introduction of potential aberrant or overcorrected data. This is in line with previous research highlighting the benefit of transformed/corrected data for multivariate methods, while warning against any such normalisation for α-diversity metrics (Lutz et al., 2022).

### 2.10 Enterotype analysis

A topic-modelling-based approach was performed, based on the methods described by Hosoda *et al.* and Breuninger *et al.* using latent Dirichlet allocation to identify microbial components (MCs) (Breuninger et al., 2021; Hosoda et al., 2020). The model was constructed using the Python genism package with the following parameters: passes= 20, iterations= 400, alpha= ‘auto’, eta= ‘auto’. The final number of MCs (*k =*4) was chosen manually based on data inspection and biological interpretation. This produced an enterosignature-like profile showing the proportion of each MC per sample, visualised as a heatmap using the scipy Python package. Finally, manual token clustering revealed the presence of six distinct enterotypes based on the aforementioned MC profiles.

### 2.11 *In vitro* fermentation

An *in vitro* batch fermentation was performed, based on a previous protocol (Uerlings et al., 2020), to test the capacity of the microbiota of animals from different farming conditions to ferment prebiotic galactooligosaccharides (commercially available as Bimuno®). Galactooligosacchardies were chosen in particular given their known degradation by bacteria such as bifidobacteria which were differentially abundant in some groups. For this, six groups, each consisting of three biological replicates were used to provide faecal inocula. For each animal whose sample was included, a 10% faecal inoculum was prepared. For this, each faecal sample was thawed at 39°C before being mechanically homogenised in sterile phosphate-buffered saline and filtered through a 20-µm-pore sieve. A fermentation buffer was prepared following a modified protocol as summarised in Table S3 (Menke and Steingass, 1988). All buffer solutions were autoclaved before use. Mucin-covered plastic microcosms were also prepared as recommended previously (Tran et al., 2016), with two added to each fermentation vial. To each vial, 0.1 g substrate was added, 8 mL fermentation buffer, and 2 mL faecal inoculum, resulting in a final faecal concentration of 2%. Vials were flushed with an anaerobic gas mixture (90% N_2_, 5% H_2_, 5% CO_2_), sealed shut, and left in an oscillating water bath at 39°C. Each group had three biological replicates for each condition.

Fermentation took place for 48 h, with gas measurements at 2, 5, 8, 12, 16, 20, 24 and 48 h using a manometer (Tracker 200, Bailey & Mackey Ltd., Birmingham, UK). Gas production parameters were analysed according to the formulae of Groot *et al.*, with maximum gas volume (A), inflection parameter (B), rate constant of gas production (C), time to reach maximum rate of gas production (T_MAX_), and maximum rate of gas production (R_MAX_) all calculated (Groot et al., 1996). For each group, the gas production was calculated after subtracting the gas production of a blank vial (no substrate, just faecal inoculum). Samples were also collected at 12 and 48 h for SCFA analysis. Final SCFA concentrations were normalised against the blank for each inoculum.

### 2.12 Statistical analysis

Statistics were calculated as described in the methods above. For all parameters, normality was first assessed via the Shapiro-Wilk test to determine the appropriate subsequent statistical test. For non-normally distributed data, as applicable for microbiota data, the Wilcoxon (two groups) and Kruskal–Wallis (three or more groups) tests were used to determine significant differences between groups. For pairwise comparisons, the Dunn’s post hoc test was employed. Unless stated otherwise, a *P*-value < 0.05 was deemed the threshold for statistical significance.

## 3 Results

### 3.1 Final cohort

**Fig. 1:**
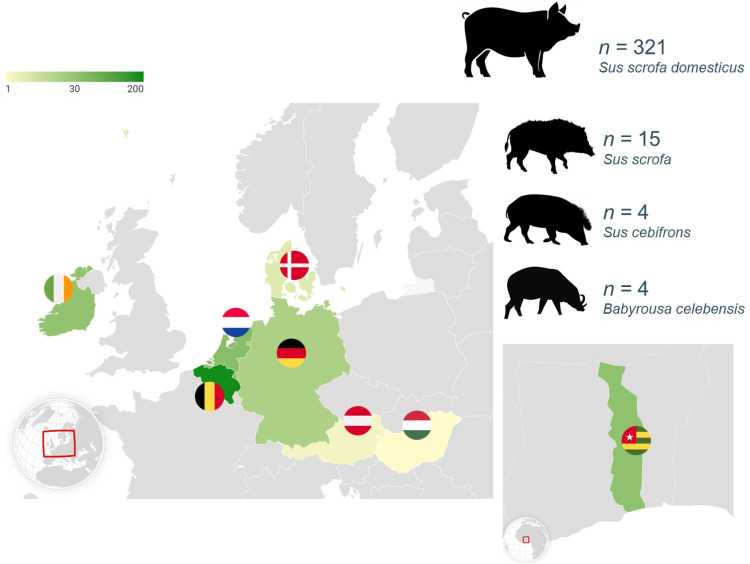
Sampling locations of animals comprising the final cohort. Within Europe, faecal samples were collected from Belgium, the Netherlands, Germany, Denmark, Austria, Hungary, and Ireland, and in Africa, from Togo. In total, 321 domestic pigs were sampled, 15 wild boar, 4 Visayan warty pigs, and 4 North Sulawesi babirusas. Each country is colour-coded according to the number of animals sampled, with dark green representing the highest number and light yellow representing the lowest number of animals.

The final cohort comprised 344 pigs and other members of the family Suidae (suids) from across seven European countries and one African country (Fig. 1). Animals came from 40 separate locations including standard commercial farms, small-scale traditional farms and smallholdings, zoos, and the natural environment (Belgian woodland). Alongside the domestic pigs, a small number of wild boar, Visayan warty pigs, and babirusas were also included in the cohort. No animals were known to have any signs of disease. Full data for each category can be found in supplementary file 1.

### 3.2 Taxonomic overview and defining a core microbiota

Across the 344 samples, a total of 121 516 ASVs were found. ASVs comprised both bacteria and archaea, and amounted to 20 phyla and 667 genera. Analysis of the cohort’s total (γ-) diversity by diversity area modelling indicated that full potential diversity had not yet been reached with 344 animals, although the number of genera did begin to increase more gradually after ∼250 samples (Figure S3).

At the level of the individual pig, the median number of genera per animal was 183 (range: 81−290). Of all genera, just seven were present in all samples: *Prevotella* (mean abundance 10.97%)*,* unclassified *Lachnospiraceae* (5.08%), unclassified *Oscillospiraceae* (4.05%), *Cryptobacteroides* (3.14%)*, CAG-83* (*Oscillospiraceae*) (1.69%)*, GCA-900199385* (*Christensenellales*) (1.11%), and *Alloprevotella* (0.97%).

A core suid microbiota was found to comprise 35 genera (Table S4). These core genera had a combined median relative abundance of 69.75%. Within this core microbiota, *Clostridium, Lactobacillus, Prevotella,* and *Treponema* displayed the highest average relative abundances. Furthermore, 13 core species were identified, fulfilling the same criterion (Table S4).

### 3.3 The role of lifestyle factors in shaping gut microbiota composition

**Fig. 2:**
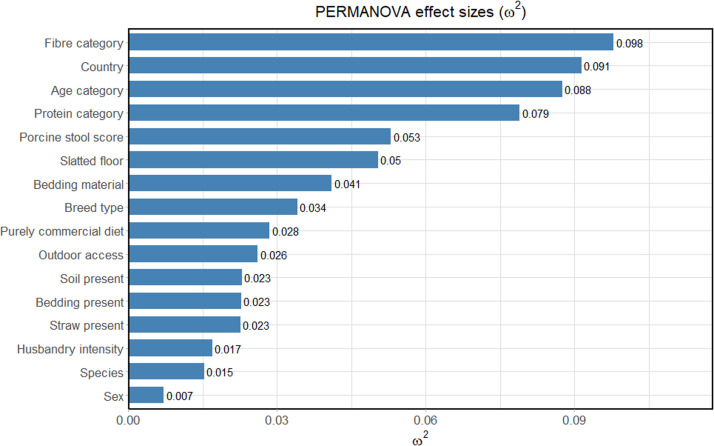
PERMANOVA ω^2^ effect sizes of lifestyle factors on microbiota composition. Lifestyle factors comprised the fibre category (low, medium, high, and very high), country, age category (lactation/nursery/growing/finishing/mature), protein category (low, medium, and high), slatted floor presence, bedding material (straw/wood shavings/wood chips), breed type (commercial/traditional/wild), purely commercial diet, outdoor access, soil presence, bedding presence, husbandry intensive (small-scale/intensive), species (domestic pig, wild boar, Visayan warty pig, and North Sulawesi babirusa), and sex (where both castrated and intact males were grouped together).

Following removal of the wild animals (15 wild boar) from the cohort, dietary crude fibre content, age category, and crude protein content had the largest ω^2^ effect sizes of 9.79%, 8.75%, and 7.90% respectively (Fig. 2). Faecal water content, categorised as ‘porcine stool score’ had the next largest effect size, followed by environmental variables including the presence of a slatted floor, and the presence of bedding material. In contrast, while still significant according to PERMANOVA, the biological, host-related variables of species and sex had the smallest effect sizes, although it should be noted that very few animals (< 3% of the cohort) were of a different species to the domestic pig. Antibiotic use was recorded but not analysed, given that only a small proportion of European pigs had been treated with antibiotics. Likewise, breed was considered (supplementary file 1), but not included in the final analysis given the complex pedigrees of many crossbred individuals. Instead, for analysis, individual breeds were grouped into the categories: commercial or traditional.

Random forest classification revealed a relatively high predictability of microbiota composition according to age category (79.6% correct classification). The lactation and mature groups were classified most accurately (3% and 9% error respectively), with the growing age group showing 20% error. Nursery and finishing pigs were however poorly distinguishable, with misclassification rates of 54% and 46% respectively. Similarly, a random forest regression model indicated that dietary crude fibre could explain 79% of variance, echoing the significant contribution of both age and diet to microbiota composition.

In terms of housing environment, the presence of a slatted floor was also demonstrated as a very strong predictor of microbiota composition, with random forest classification correctly identifying 93% of animals based on this variable alone.

### 3.4 Investigating the associations between metadata variables

Multiple correspondence analysis and Cramér’s V test revealed significant associations between many metadata variables (Figures S4–5). Feeding of a non-purely commercial diet was for instance very strongly associated with outdoor access (*V* *=*0.91), as well as showing a strong association with slatted floor presence (*V* *=*0.71). In short, animals raised in less-intensive, traditional systems were generally more likely to have exposure to bedding material, and a non-commercial diet, whereas animals in more intensive commercial systems were likely to have slatted flooring, a purely-commercial diet, and no access to soil or the outdoors.

Noteworthily, χ^2^ and Cramér’s V tests confirmed a strong, significant association between age category and fibre category, and age category and protein category (χ^2^=282.94 and 211.02, *V* *=*0.74 and 0.79 respectively, and *P* < 0.001 for both).

### 3.5 Age category and the microbiota

**Fig. 3:**
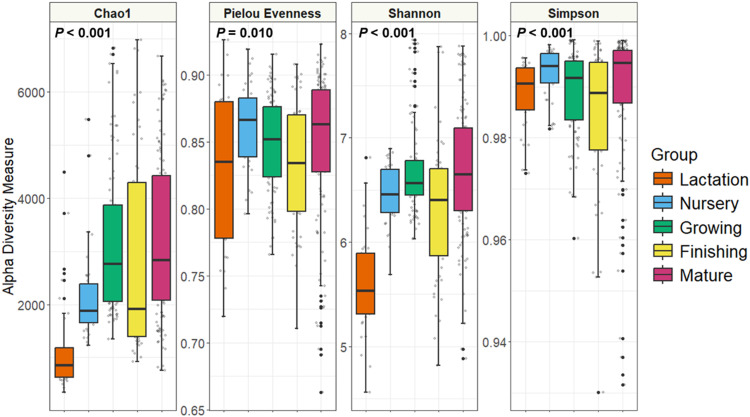
α-Diversity by age category. Microbial diversity is shown for the Chao1, Pielou evenness, Shannon, and Simpson indices for each age category. Statistical differences were determined according to the Kruskal-Wallis test.

For all indices measured, α-diversity was significantly different among the cohort according to age category (Fig. 3). For the Chao1 and Shannon indices in particular, the lactation group exhibited lower α-diversity. Generally, an increase in α-diversity was observed as age increased among all age categories, with the exception of reduced diversity among the finishing group.

**Fig. 4:**
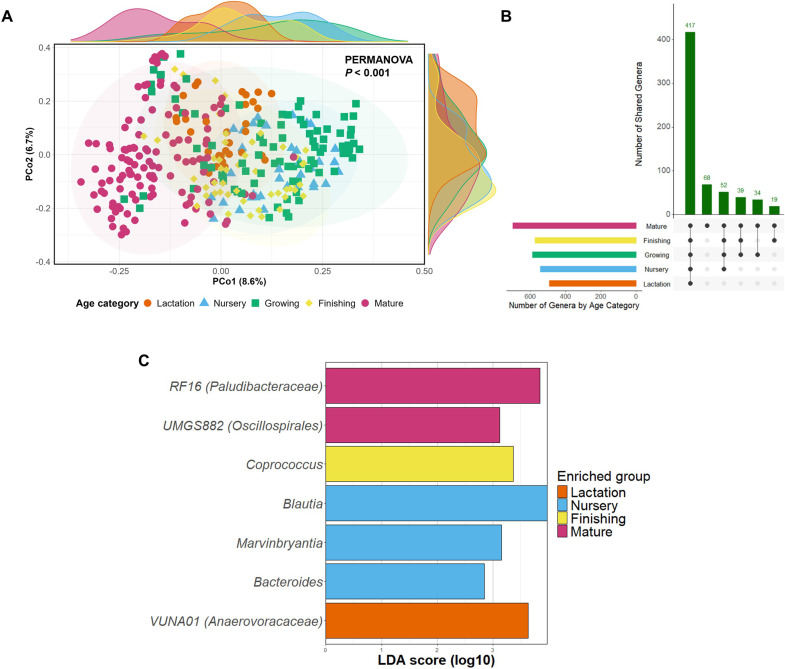
The impact of age category on the composition of the microbiota. PCoA of β-diversity was performed using Bray-Curtis distance (A). Age category was deemed significantly associated with β-diversity according to PERMANOVA. The number of shared genera between age categories are also plotted as an UpSet plot (B). Shared genera < 10 were not visualised. Differentially abundant genera according to LEfSe analysis (LDA > 2.5) are plotted as a bar plot (C).

For β-diversity, PCoA revealed some clustering according to age category, with mature pigs forming a distinct cluster (Fig. 4A). The other age categories however were less distinguishable, although the first two components visualised just 15.3% of overall variation.

Integrating taxonomy, all age categories shared 417 genera in total, with an additional 68 appearing exclusively among mature pigs (Fig. 4B). LEfSe analysis revealed a small number of genera to be associated with different age categories (Fig. 4C).

### 3.6 Diet, faecal water content, and housing environment

#### 3.6.1 Crude fibre and protein

Dietary crude fibre and protein demonstrated varied influences on the microbiota. Generally, increased crude fibre led to significantly increased α-diversity (*P <*0.001) (Figure S6). Conversely, increased crude protein resulted in decreased α-diversity (Chao1 and Shannon) (*P <*0.001), while showing the opposite trend for Pielou evenness (*P* *=*0.008) (Figure S7). For taxonomy, LEfSe analysis also revealed some inverse trends for fibre and protein, with *RF16* (*Paludibacteraceae*) and *Cellulosilyticum* associated with diets very high in fibre and low in protein, and *Collinsella* with high protein and low fibre (Table 1).

**Table 1:** Genera Associated with Different Dietary Fibre and Protein Levels.

	**Fibre**	**Protein**
**Very high**	*RF16* (*Paludibacteraceae*), *Cellulosilyticum, Clostridium, Ruminococcus*	−
**High**	*Intestinibacter*	*Escherichia-Shigella, Collinsella, Phascolarctobacterium, Bacteroides, Blautia*
**Medium**	*Blautia, Coprococcus*	*Butyricicoccus, Roseburia, Mitsuokella,* unclassified *Lachnospiraceae*
**Low**	*Collinsella*	*Terrisporobacter, RF16* (*Paludibacteraceae*), *Cellulosilyticum*

LEfSe analysis was performed to determine genera significantly associated with dietary fibre and dietary protein categories. Genera with an LDA value > 2.5 were deemed statistically significant.

#### 3.6.2 Faecal water content

Increased water content was associated with significantly reduced α-diversity across the six porcine stool scores (*P <*0.001). Porcine stool score was also shown to have a significant impact on β-diversity, with some clustering visible via PCoA (Figure S8). The exact water content percentage was then assessed for finer resolution, which showed significant correlations with many genera. *Bacillus, Cellulosilyticum, Cryptobacteroides, Romboutsia, Treponema,* and *Akkermansia* were among the genera negatively correlated with faecal water content, while *Blautia, Dialister, Lactobacillus, Megasphaera, Prevotella, Streptococcus,* and *Faecalibacterium* were positively correlated with water content (ρ ≥ 0.32, *P*_Bonferroni_ *≤*0.008 for all).

#### 3.6.3 Housing system and husbandry

Several housing system variables were shown to have significant impacts on α-diversity. Firstly, the presence of a slatted floor in the pig’s pen/enclosure was associated with significantly lower α-diversity for all four metrics (*P <*0.001) (Figure S9). On the contrary, pigs with outdoor access were associated with significantly higher α-diversity (Pielou evenness, Shannon, and Simpson indices) (*P <*0.001) (Figure S10). Likewise, the presence of soil in the animal’s environment was associated with higher diversity for the Pielou evenness and Simpson indices, while being associated with significantly lower Chao1 diversity (*P <*0.001) (Figure S11). In contrast, neither the presence of bedding material, nor more specifically, the presence of straw, were associated with significant differences in α-diversity. However, while Togolese pigs consistently exhibited higher α-diversity than their European counterparts (*P <*0.001), none were provided with bedding material. Reanalysis on just the European cohort showed that the presence of straw was associated with a significant increase in their α-diversity for all indices (*P ≤*0.009), with bedding material in general following suit, except for the Simpson index (Figures S12–13).

#### 3.6.4 Rustic and conventional husbandry

All of the environmental and husbandry variables were shown to have a significant impact on β-diversity (*P <*0.001). Nevertheless, many of these covaried across the cohort, as highlighted further by the PCoA (Figure S8), in line with the aforementioned multiple correspondence analysis and Cramér’s V test.

Taxonomically, LEfSe analysis further emphasised the considerable overlap between husbandry variables. Overall, more “rustic” farming styles (traditional, smaller-scale husbandry, the lack of a slatted floor, farming of traditional breeds, and the feeding of a non-commercial diet) were associated with *Treponema, RF16, Cryptobacteroides, Cellulosilyticum, Turicibacter,* and *Romboutsia*. On the other hand, the presence of a slatted floor, the rearing of commercial breeds, and feeding of a purely commercial diet defined a more “conventional”, intensive farming system. This was characterised by increased *Prevotella, Lactobacillus, Limosilactobacillus, Megasphaera, Blautia, Gemmiger, Streptococcus,* and *Butyricicoccus.*

Given the observed association between the husbandry variables, and their common microbial impacts, the farming systems will hereafter be referred to as ‘rustic’ and ‘conventional’ when appropriate to refer to the generalised systems over specific variables.

#### 3.6.5 Disentangling environment from diet

**Fig. 5:**
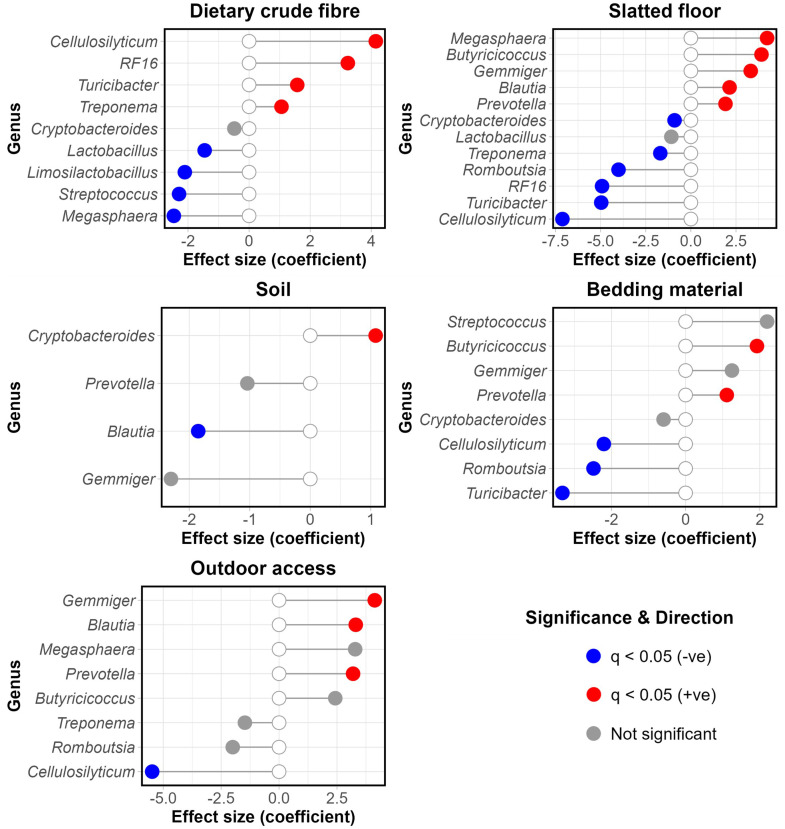
Lollipop plots of MaAsLin2 output for genera of interest with dietary and environmental factors. MaAsLin2 output was plotted for the genera *Treponema, Cryptobacteroides, Cellulosilyticum, Turicibacter, Romboutsia, RF16, Prevotella, Lactobacillus, Limosilactobacillus, Megasphaera, Blautia, Gemmiger, Streptococcus,* and *Butyricicoccus*. FDR correction was applied to produce *q*-values. Variables with negative coefficients were coloured in blue, positive in red, and those with non-significant *q*-values after FDR correction in grey. *N=*171.

Despite LEfSe analysis highlighting said genera as common to a range of environmental variables, the diet (namely crude fibre) equally covaried with the environment. In general, rustic farming was more likely to be accompanied by diets higher in fibre. As such, for a subset of pigs for which exact dietary crude fibre was known (*n* *=*171), the multivariate test MaAsLin2 was used and subsequently revealed these genera to have complex relationships with both the environment and diet (Fig. 5).

MaAsLin2 signalled *Cellulosilyticum, RF16, Turicibacter,* and *Treponema* as significantly associated with increased crude fibre (*q <*0.001, < 0.001,= 0.009, 0.035 respectively). Nevertheless, these genera also had independent associations with slatted flooring (rather, the lack thereof), and outdoor access (in the case of *Cellulosilyticum*). Curiously, while the presence of a slatted floor and the provision of bedding material demonstrated a strong negative association (φ= −0.78, χ^2^= 100.90, *P <*0.001), *Prevotella* and *Butyricicoccus* were positively associated with both factors, and *Cellulosilyticum, Romboutsia,* and *Turicibacter* negatively so for both (Fig. 5). Further analysis revealed that even at the species level, the same trend was observed with species such as *Prevotella* spp. *017347605* being positively associated with both bedding material and slatted floor presence.

Of note, *Dorea, Oribacterium,* and *Faecalibacterium* were also among the genera associated positively with outdoor access, while *Akkermansia* showed a negative association (all *q <*0.001).

### 3.7 Predicted microbial functionality

PICRUSt2 predicted a number of pathways with significantly different abundance between groups for the aforementioned rustic- and conventional-associated lifestyles (*P*_Bonferroni_ *<*0.001 for all). Of note, the lack of a slatted floor was associated with fermentation pathways including the fermentation of pyruvate to butyrate and propionate, acidogenic fermentation, and mixed acid fermentation. Amino acid (L-leucine, L-tyrosine, and L-histidine) degradation was also predicted to be higher in the pigs without slatted floors. Similarly, the presence of straw was also associated with increased L-leucine degradation and butyrogenic fermentation.

### 3.8 Enterotyping

**Fig. 6:**
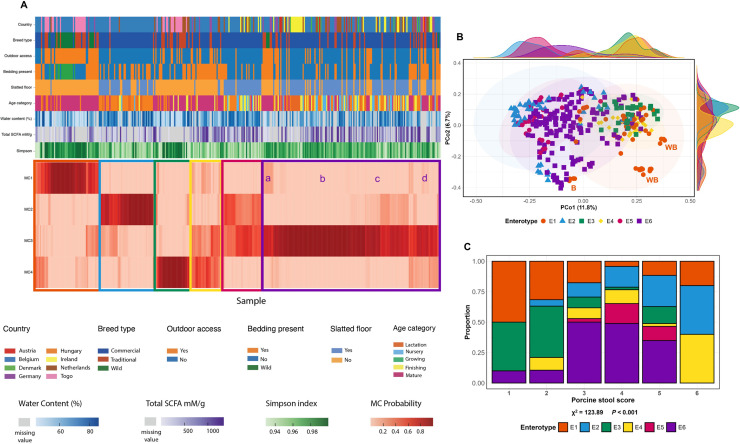
Enterotype profiles and their distribution according to various metadata. The probability of MC distributions are visualised for each sample in figure A, alongside their respective metadata: country, breed type, outdoor access, bedding presence, slatted floor, age category, faecal water content, total SCFAs, and Simpson index (α-diversity). Four distinct subgroups indicating some variation in terms of their MC probabilities within enterotype E6 are indicated with the letters a, b, c, and d. The distribution of enterotypes are also visualised by PCoA (B) according to Bray-Curtis distance. As distinct outliers, two clusters of wild boar are denoted with the letters WB, and a cluster of babirusas are indicated with the letter B. The distribution of porcine stool score is also demonstrated for each enterotype (C), which was deemed significant according to the χ^2^ test.

Following the reincorporation of the wild animals into the cohort, LDA revealed distinct enterosignature profiles consisting of four MCs differing in their proportions of ten genera (Figure S14). From these MCs, token clustering determined the presence of six enterotypes, distinct in terms of their enterosignature profiles, hereafter referred to as E1 – 6 (Fig. 6)

Within each enterotype, some commonality between lifestyle metadata was visible, as summarised in Table 2. E1 represented the natural environment, and small-scale farming conditions, consisting of the wild boar, babirusas, some of the Togolese pigs, and European pigs reared in small-scale facilities with bedding material. These animals belonged largely to the mature age category, and generally had higher α-diversity. This enterotype exhibited increased *Clostridium,* as well as relatively high levels of *Prevotella* and *Terrisporobacter*. Of note, as evidenced by PCoA, the members of E1 were visibly spread out, with the wild boar and babirusas appearing particularly distinct (Fig. 6).

**Table 2:** Microbial and lifestyle characteristics for each enterotype.

**Enterotype**	**Microbial component/s**	**Proportion of cohort**	**Characteristic genera**	**Constituent characteristics**
**E1**	MC1	15.7% (*n=*54) *N* locations= 15	*Prevotella, Cryptobacteroides*	Wild boar (wild*,* Belgium), babirusas, Togolese pigs, pigs from zoos and small-scale farms. Mainly mature animals.
**E2**	MC2	14.2% (*n=*49) *N* locations= 8	*Streptococcus, Prevotella, Lactobacillus*	Pigs from standard commercial settings. Mainly growing animals.
**E3**	MC4	8.1% (*n=*28) *N* locations= 7	*Lactobacillus, Clostridium, Prevotella, Cryptobacteroides*	Togolese pigs, Belgian and Dutch pigs from small-scale farms (straw provided). Mainly mature animals.
**E4**	MC3 and MC4	8.4% (*n=*29) *N* locations= 9	See MCs 3 and 4	Pigs in conventional facilities (with bedding material or outside access). Mainly mature animals.
**E5**	MC2 and MC3	9.3% (*n =*32) *N* locations= 8	See MCs 2 and 3	Pigs in conventional facilities (some with and some without bedding). Mixed ages.
**E6**	MC3	44.2% (*n =*152) *N* locations= 23	*Clostridium, Prevotella, Terrisporobacter*	Most diverse; wide range of animals: conventional commercial facilities to pigs outside and zoo-reared pigs and Visayan warty pigs → possible ‘sub-enterotypes’ Mixed ages.

While broadly similar to E1 in terms of microbial composition and animals’ metadata, E3 exhibited an increased probability of the lactic acid bacteria *Lactobacillus* and *Limosilactobacillus*. E2, E4, and E5 comprised animals from more conventional facilities. Across these three enterotypes, α-diversity was relatively lower, although taxonomy differed between the three. E2, with markedly increased MC2, was characterised by *Streptococcus, Prevotella,* and *Lactobacillus*. E4 on the other hand was characterised by both MC3 and MC4, while E5 exhibited increased MC2 and MC3 (Table 2).

E6 was the largest enterotype, comprising 44.2% of the cohort, and was the most diverse in terms of the animals’ lifestyle. Broadly speaking, *Terrisporobacter, Clostridium,* and *Prevotella* served as hallmarks of E6. Nevertheless, MC probabilities suggested the presence of relatively distinct subgroups, possibly four ‘sub-enterotypes’ (denoted a – d, Fig. 6). E6a had an increased probability of MC1, and consisted of zoo-reared pigs and Visayan warty pigs. The other subgroups however displayed little similarity between the lifestyle metadata of their constituent animals.

Enterotype was shown to be significantly associated with the porcine stool score as a marker of water content (χ^2^= 123.89, *P <*0.001). Reduced faecal water (stool scores 1 and 2) were associated more closely with E1 and E3, while increased water content was associated with E2. E5 and E6 comprised samples primarily with faecal scores 3–5 (Fig. 6).

### 3.9 Mapping the interplay between the microbiota, diet, and volatile fatty acids

Animals with low fibre diets had significantly reduced total faecal VFAs and SCFAs (*P* *=*0.039 and 0.029 respectively). While total VFAs and SCFAs were raised for the medium, high, and very high categories, the very high cohort had the highest acetate compared to the other groups (*P* *=*0.020), yet significantly reduced butyrate and valerate (*P* *=*0.004 and 0.007 respectively). The low fibre group also had significantly elevated MCFAs (*P <*0.001). Protein category showed the opposite trend, with the low protein cohort having significantly elevated total VFAs and SCFAs (*P* *=*0.001 and 0.002 respectively), alongside reduced MCFAs (*P* *=*0.003). Neither fibre nor protein groups showed significant differences for BCFA concentrations.

The husbandry variables of slatted flooring, bedding material presence, and breed type did not show significant differences for VFA concentrations. Nevertheless, the group receiving a purely commercial as opposed to non-commercial diet was shown to exhibit significantly elevated total VFAs, SCFAs, MCFAs, and BCFAs (*P* *=*0.042, 0.013, 0.032, and 0.012 respectively). However, while acetate, propionate, and caproate were elevated in the commercial diet group (*P* *=*0.038, < 0.001,= 0.012 respectively), there were no significant differences for butyrate or valerate.

**Fig. 7:**
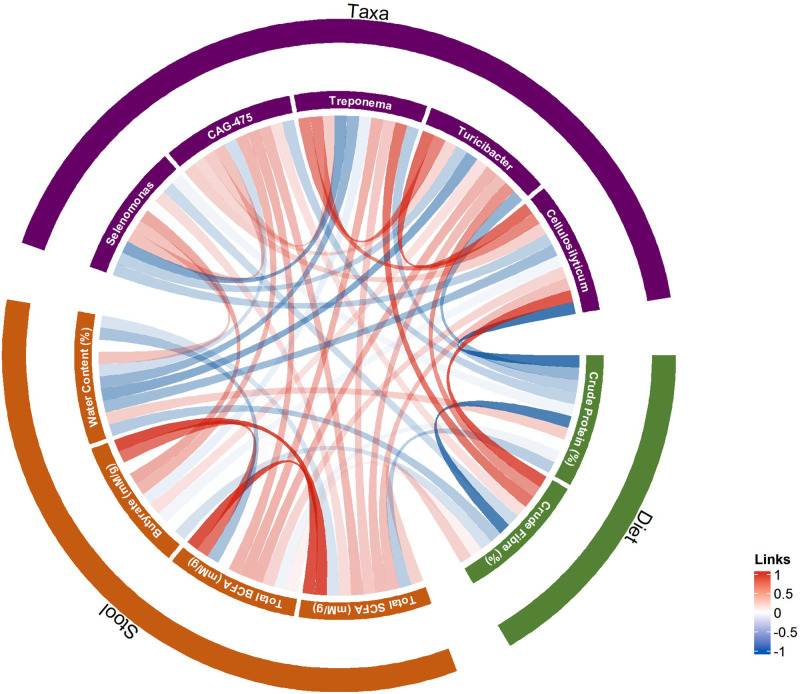
Chord diagram of correlations between dietary factors, stool parameters, and taxa. Dietary factors comprise crude protein and fibre. Stool parameters consist of total SCFAs, total BCFAs, butyrate, and faecal water content. Correlations were calculated according to the Spearman rank test, with positive correlations visualised in red, and negative in blue. Insignificant correlations (*P>*0.05) were not visualised.

Correlation analysis revealed *Selenomonas* to have the highest positive correlation with faecal butyrate (ρ= 0.35, *q <*0.001). *Selenomonas* also exhibited a negative correlation with *Treponema* and crude fibre, but a positive correlation with faecal water content (Fig. 7). For total faecal VFAs, *CAG-475* (*Christensenellales*) had the highest positive correlation (ρ= 0.39, *q* < 0.001). *CAG-475* was also positively correlated with crude fibre and negatively so with faecal water content (Fig. 7).

Several of the aforementioned husbandry-associated genera from LEfSe analysis revealed weak, yet statistically significant correlations with VFA levels. *Treponema, Cellulosilyticum, Turicibacter,* and *Romboutsia*, all associated with rustic husbandry, were positively correlated with acetate (all ρ > 0.20, *q ≤*0.014), and for *Treponema* and *Turicibacter*, also with propionate (ρ > 0.20, *q ≤*0.012). Equally, *Treponema, Cellulosilyticum, Turicibacter,* and *Romboutsia* were positively correlated with isobutyrate (ρ ≥ 0.23, *q ≤*0.005), and *Treponema* and *Cryptobacteroides* with total BCFAs (ρ= 0.33 and 0.29, *q <*0.001 and= 0.007 respectively). In contrast the genera associated with conventional husbandry did not show positive correlations with any VFA, with the exception of *Gemmiger*, which showed a single weak positive correlation with the MCFA caproate (ρ= 0.19, *P* *=*0.029).

### 3.10 *In vitro*fermentation

**Fig. 8:**
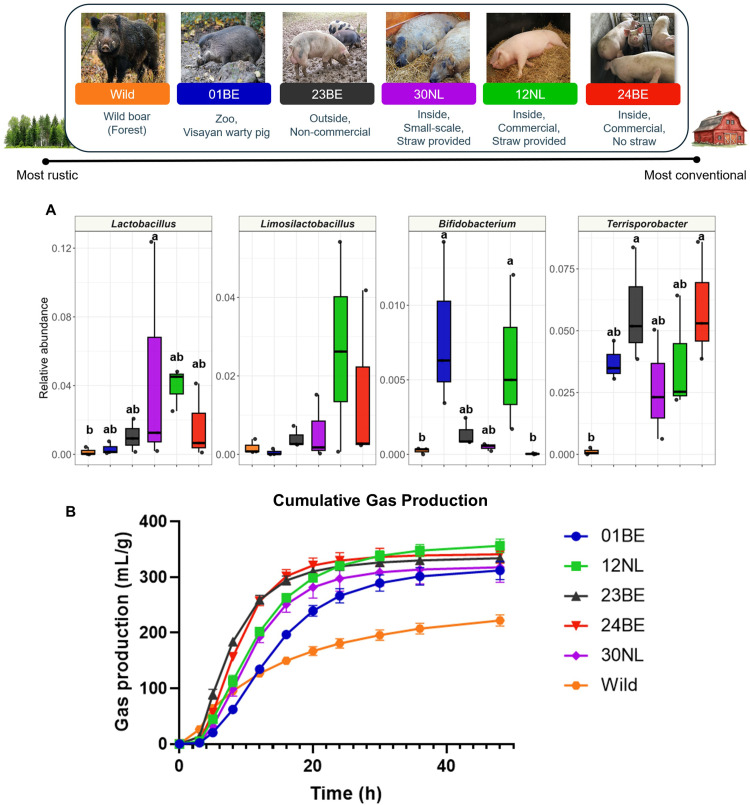
Relative abundances of selected genera among fermentation donor groups and gas production from galactooligosaccharide fermentation. The lifestyle characteristics of each microbiota donor group are visualised in the legend above. Each group consisted of *n=*3 biological replicates for each substrate. Relative abundance of the lactic acid bacteria *Lactobacillus, Limosilactobacillus,* and *Bifidobacterium,* alongside *Terrisporobacter* are shown for each group as boxplots (A). Dunn’s post hoc test with FDR correction was performed for pairwise comparisons, with differing letters denoting statistically significant differences between groups. Cumulative gas production from the fermentation of galactooligosaccharides is plotted over 48 h, with the error bars showing the standard error of the mean (B).

Finally, an *in vitro* fermentation using a small subset of the cohort was performed to investigate the link between *in silico* microbial classification and *in vitro* functionality. Despite galactooligosaccharides being known to be degraded primarily by bifidobacteria and lactobacilli (Grimaldi et al., 2016), the present *in vitro* results revealed a more complex and dynamic role of other bacteria. *Bifidobacterium,* a key galactooligosaccharide-fermenter, was increased in groups 01BE and 12NL compared to the wild group and 24BE (Fig. 8A). However, the parameter B indicated that fermentation began more slowly for the 23BE pigs compared to those from the wild group, 01BE, and 12NL (Table S5). Nevertheless, pigs from both 23BE and 24BE had significantly higher maximum rates of gas production than all other groups, despite contrasting *Bifidobacterium* abundances, and husbandry systems.

Correlation analysis of the starting microbiota and gas parameters revealed R_MAX_ to be positively correlated with *Clostridium* (ρ= 0.74, *q* *=*0.047)*,* and *Terrisporobacter* (ρ= 0.72, *q* *=*0.047). Of note, *Terrisporobacter* was significantly increased among 23BE and 24BE (Fig. 8A). In contrast, R_MAX_ was negatively correlated with genera including *Parabacteroides* (ρ= −0.75, *q* *=*0.043), and *Lawsonibacter* (ρ= −0.71, *q=*0.047). Neither *Bifidobacterium* nor *Lactobacillus* exhibited significant correlations.

At 12 h of fermentation, acetate production was significantly increased in the 12NL fermentation vials compared to the 23BE and wild groups (Table 3). At this stage, there was no propionate production. Butyrate production was minimal, although was increased in 30NL and 12NL. SCFA measurements at the end (48 h) of the fermentation revealed the wild group to have significantly lower acetate production compared to 01BE, 12NL, and 30NL which had the highest. On the other hand, propionate production was significantly increased among the wild group and 23BE.

**Table 3:** Acetate, propionate, and butyrate production after 12 and 48 h of galactooligosaccharide fermentation.

	**Group**	**Acetate**		**Propionate**		**Butyrate**	
		Mean	SEM	Mean	SEM	Mean	SEM
12 h	**01BE**	16.73^ab^	2.22	0.00	0.00	0.00^a^	0.00
	**12NL**	41.02^a^	3.43	0.00	0.00	5.50^b^	0.90
	**23BE**	14.65^b^	1.85	0.00	0.00	2.90^ab^	0.06
	**24BE**	34.42^a^	5.85	0.00	0.00	2.35^ab^	1.19
	**30NL**	30.83^ab^	5.49	0.00	0.00	7.49^b^	3.37
	**Wild**	12.47^b^	3.03	0.00	0.00	0.00^a^	0.00
	***P*-value**	**0.039**	NA	**0.027**			
**48** h	**01BE**	54.32^a^	3.08	7.59^abc^	3.44	22.59^ab^	4.20
	**12NL**	57.20^a^	0.95	7.64^ab^	0.17	18.58^ab^	0.70
	**23BE**	50.87^ab^	10.89	32.37^ac^	7.36	22.80^ab^	8.09
	**24BE**	53.03^ab^	11.68	9.97^abc^	1.28	58.98^a^	3.45
	**30NL**	54.61^a^	3.93	0.00^b^	0.00	17.36^b^	5.96
	**Wild**	19.76^b^	6.80	29.78^c^	3.15	24.95^ab^	5.00
	***P*-value**	0.162	**0.018**	0.165			

Kruskal–Wallis tests were used to determine significant differences across the groups, with Dunn’s post hoc test for pairwise comparisons. Differing superscripts denote statistically significant differences (*P <*0.05) between groups. Each group consisted of *n* = 3 biological replicates.

## 4 Discussion

The present study investigated changes in the porcine microbiota over a gradient of diverse lifestyles, understanding which factors are the most important, and exploring what taxonomic changes may mean for microbial metabolites and functionality. Accordingly, the final dataset represents a range of varied microbiotas, across two continents, shaped by a diverse spectrum of lifestyles and diets, amounting to one of the most diverse single-study porcine cohorts to date.

While a relatively large core microbiota was found, around 30% on average of each individual’s taxonomic abundance was not incorporated within this core. Furthermore, diversity area modelling suggested that 667 genera would have been surpassed when studying a larger cohort. While increased samples would have likely reduced the core microbiota, the present dataset is approximately on par with human studies finding median core microbiota abundances of 72.2%, despite human populations being renowned for high lifestyle and dietary diversity (Falony et al., 2016).

Assessing β-diversity through PCoA should be approached with prudence given that the first two principal coordinates described just 18.5% variation in the present study. Nevertheless, the wild boar appeared as visibly distinct outliers from the majority of the cohort, with two separate clusters even amongst the wild boar themselves. This distinction of wild animals from their domesticated/captive counterparts has indeed been highlighted by a number of other porcine/suid studies (Correa-Fiz et al., 2019; Kuthyar et al., 2023; Petrelli et al., 2022; Rahman et al., 2024).

Of all factors, age category and proximate dietary composition exhibited the largest impacts on the microbiota, in line with previous research (Han et al., 2018; Wang et al., 2019). Country also exhibited a high effect size, although given its frequent confounding with farming system types, which has also been a complication in porcine meta-analyses (Dong et al., 2023), we deemed this of less importance rather than a genuine geographical driver of microbial differences. Although some differences were found in the microbial impacts of age and fibre categories, this is likely in part due to the greater resolution of age compared to relatively broad fibre categories. Furthermore, given the varied dietary regimens of the current cohort, there may have been less overlap between age category and proximate dietary composition. Largely however, age category and diet proved to be strongly interconnected, with older pigs having diets richer in fibre and reduced in protein, particularly in the case of conventional farming.

Despite the significance of age category, the current data present a multifaceted picture, with environmental variables likewise conveying significant impacts. Previous studies emphasised the importance of lifestyle and rearing conditions as a whole in shaping microbial composition, yet beyond diet, studies looking at particular environmental variables have yielded conflicting results (Holman et al., 2023; Kubasova et al., 2017; Megahed et al., 2019; Papić et al., 2025; Wen et al., 2021). The present data suggest two broad farming types associated with the microbiota: those that employ more traditional, rustic practices, and in contrast, more standardised, conventional farming systems. Within these two categories, it remains challenging to distinguish between specific factors given that animals with each lifestyle type were more likely to have multiple factors typical of that particular lifestyle. Nevertheless, the individual factors within these broad lifestyle types consistently revealed a number of genera in common. Rustic farming was associated with genera including *Treponema, Cryptobacteroides, Cellulosilyticum,* and *Turicibacter,* some of which were correlated with increased faecal VFAs. These genera have all been shown to be associated with increased fibre consumption, or possess fibre-degrading enzymes (Angelakis et al., 2019; Cai et al., 2010; Niu et al., 2015; Rühlemann et al., 2024; Wu et al., 2022; Xu et al., 2024). While there was some overlap between the environment and fibre consumption, multivariate MaAsLin2 did suggest some unique contribution of environmental factors in promoting the abundance of these genera.

Bedding material likely also increases fibre consumption, as evidenced by clear remnants of straw in the faecal samples of the animals housed with straw in this study. *Cryptobacteroides* in particular, also showed no significant association with crude fibre. *Cryptobacteroides* has previously been studied in ruminant ungulates, and has been shown to be a marker of rural, African human populations, although its influences and roles remain poorly classified in pigs (Rühlemann et al., 2024; Xu et al., 2024). Recent enterosignature profiling nevertheless showed it to co-occur with *RF16* (Vourlaki et al., 2025)*,* another genus shown to be associated with rustic farming in the current study. Previous research has analysed the impact of soil exposure, and bedding material in driving α-diversity and taxonomy, but did not find an association with these bacteria, instead demonstrating a significant association with *Prevotella* (Megahed et al., 2019; Vo et al., 2017; Wen et al., 2021).

Of note, *Prevotella* showed a complex association in the current dataset, being indeed associated with the presence of bedding material, while equally being associated with factors typical of conventional farming such as purely-commercial diets and slatted flooring. A previous human study investigating the microbiota of Filipino children found *Prevotella* to be positively correlated with dietary carbohydrate intake (Nakayama et al., 2017). Therefore, this complex trend may be attributable to both increased fibre via bedding material among rustically-farmed pigs, and increased starchy carbohydrates in the commercial diets of conventionally-farmed pigs housed on slatted floors. It should be noted however that said taxonomic trends should not be overinterpreted in terms of functionality. Given that many species and strains within the same genus show contrasting properties, such differences should be interpreted with prudence. Such contrasts have previously been demonstrated, with some species of *Prevotella* increasing while others decrease during the post-weaning period (Ortiz Sanjuán et al., 2024). While PICRUSt2-derived predictions were also considered in the present study, such methodologies have significant limitations in terms of their accuracy. Further experimental validation would therefore be useful to determine the exact microbial functionality of the genera associated with both husbandry types.

Conversely, *Lactobacillus, Limosilactobacillus, Megasphaera, Blautia, Gemmiger, Streptococcus,* and *Butyricicoccus* were shown to be more indicative of conventional farming. The provision of starchy, cereal-based commercial feeds has been shown to upregulate genera such as *Lactobacillus* which are less abundant in wild suid populations (Ushida et al., 2016). While many pigs farmed rustically in the present study were fed with commercial feed, it tended often to be supplemented with other unprocessed grains or fresh vegetables, and animals were often living on and ergo consuming bedding material. As such, the pigs in more conventional farms likely had less diverse substrates for their microbiota, hence leading to taxonomic differences and reduced α-diversity. As evidenced in human studies, reduced dietary diversity can lead to increased fast-growing saccharolytic bacteria, and decreased α-diversity (Vermeulen et al., 2025).

Interestingly, the inclusion of the African pigs further demonstrates the complex and often compensatory role of various factors. Despite approximately half of the Togolese pigs receiving regular antibiotics, and none being provided with bedding material, they consistently had higher α-diversity than their European conspecifics. This thus highlights the compensatory effects of a diverse, fibre-rich diet, as was the case for the Togolese pigs, in what would otherwise appear to be microbiota-depleting living conditions.

As a field study, the sizes of individual groups adhering to certain variables was not controlled for. While we endeavoured to find animals with as many differences as possible, certain variables, such as conventional farms in certain countries, were nevertheless underrepresented, potentially leading to statistical biases when analysing certain factors. Future studies could potentially focus on fewer factors to better equilibrate group sizes, although this may remain challenging in a real-world setting.

Previous porcine enterotyping has relied heavily upon JSD-PAM clustering, which typically yields a smaller number of enterotypes (Hosoda et al., 2020). Using LDA-based clustering as a novel application in the porcine field, we identified six distinct enterotypes at a higher level of resolution with greater differentiation between microbial communities. These enterotypes proved highly reflective of husbandry and environmental factors, and further emphasised these factors’ importance in shaping microbial composition. Previous porcine enterotypes have flagged *Treponema* and *Prevotella* as particularly important in defining enterotypes, as was the case in the current study, albeit alongside other genera (Ke et al., 2019; Larzul et al., 2024; Mach et al., 2015; Ramayo-Caldas et al., 2016). Of all enterotypes, E6 showed the greatest number of animals, and indeed variation among those constituent animals. This may indicate that this enterotype is the most prevalent in the general pig population in terms of general microbial taxonomy, while differences in α-diversity and hence less-abundant taxa drive subtle variation. E1 also demonstrated increased diversity on the PCoA, with the wild boar and babirusas appearing visibly distinct. Babirusas are known to have exceptional digestive anatomy within the Suidae family, with rumen-like stomachs encouraging foregut fermentation (Leus and MacDonald, 1997), potentially leading to microbiota differences. Yet despite the taxonomic differences from the babirusas, and wild boar within E1, increased α-diversity, decreased water content, and *Prevotella* and *Cryptobacteroides* (with reduced lactic acid bacteria) likely defines this enterotype above all.

While porcine work traditionally focuses on stool consistency in the context of disease or microbial dysbiosis, human studies have shown a considerable relationship between water content, gut transit time, and microbiota composition (Procházková et al., 2023; Vandeputte et al., 2017). Specifically, *Akkermansia,* and methanogens such as *Methanobrevibacter* are believed to delay gut transit time via their production of methane, which in turn increases proteolytic fermentation in the colon and leads to the accumulation of potentially noxious substances (Vandeputte et al., 2017). This finding was confirmed in the present study for *Akkermansia*, although other genera such as *Cryptobacteroides, Romboutsia, Blautia,* and *Lactobacillus* showed higher correlations. The finding of the well-known butyrate-producer *Faecalibacterium* being positively correlated with water content may also reaffirm the notion from human studies of increased water content being better for gut health.

Faecal VFA concentrations further demonstrate a complex association between lifestyle and microbial metabolites. Overall, fibre was shown to be positively correlated with VFAs, although, as with water content, the very high fibre category showed slightly different trends, with higher acetate yet decreased butyrate. Many of the rustic-associated bacteria also demonstrated positive correlations with individual SCFAs, while in contrast, a conventional, commercial diet was also associated with increased VFAs. This may reflect the potentially more robust nature of the rustic-associated genera to degrade diverse dietary fibres, while equally, the starch-rich, homogenous commercial diet promotes efficient and quick fermentation. Previous pig studies have found contrasting taxonomic results, with *Prevotella* and *Lactobacillus* being positively correlated with butyrate (Sebastià et al., 2024). This may however reflect those pigs’ conventional rearing, and thus lack of overall variation in the microbiota and diet. Moreover, the negative correlation of water content and SCFAs in the present study may be indicative of gut transit time, with animals with less faecal water (and thus longer gut transit times) reabsorbing more SCFAs, or the SCFAs being converted into other metabolites as has been shown in human studies (Procházková et al., 2023; Waseem et al., 2022). Increased gut transit time in humans has also been characterised by increased proteolytic processes and an accumulation of BCFAs (Procházková et al., 2023). This was in accordance with the present study. Additionally, given the evident large variability in faecal water content, bacterial load may be an interesting variable to take into account in future such analyses considering its importance being highlighted in the human microbiota field (Nishijima et al., 2025).

Finally, an *in vitro* fermentation was performed to test whether *in silico* predictions could be translated into functional outcomes. Galactooligosaccharides were chosen in particular given their known degradation by specific bacteria, namely *Bifidobacterium* and *Lactobacillus* (Grimaldi et al., 2016; Tzortzis et al., 2005). Microbiota donors were also chosen to encompass a range of rustic to conventional settings. Two donor groups (01BE and 12NL) had significantly increased *Bifidobacterium* in comparison with the wild boar. Yet despite few starting differences in lactic acid bacteria, fermentation profiles showed 23BE and 24BE to be better adapted to efficient galactooligosaccharide fermentation. This varied functionality is further exemplified by the SCFA results which showed 23BE to have lower acetate production at 12 h, below even the wild group which ultimately had significantly reduced gas production. Yet by 48 h, 23BE had significantly increased propionate, while 24BE had increased butyrate.

Accordingly, these data suggest that alternative genera such as *Terrisporobacter,* and *Clostridium* may be more important in the fermentation of such dietary substrates. *Terrisporobacter* could for example have a similar ecological niche and function to *Lactobacillus*, with previous nutritional studies finding both *Lactobacillus* and *Terripsorobacter* increased in response to a high-amylose cornstarch diet, as well as probiotic *Lactobacillus* supplementation reducing *Terrisporobacter* abundance (Wellington et al., 2025; Zhang et al., 2023). Curious as well was the insignificant impact of *Bifidobacterium* with R_MAX_, indicating that it was likely functionally redundant. The current fermentation was carried out on just a small subset of the cohort, likely therefore not capturing all possible interindividual variation. Future studies including a greater number of individuals (biological replicates) would therefore be of value to be able to make assumptions more applicable to a larger porcine population. Nevertheless, these results highlight the complexity of predicting functionality from *in silico* taxonomy. Importantly, our data suggest that feeding pigs with known prebiotics, or implementing other microbiota-targeted interventions, may result in different effects on different hosts, not predictable purely from the abundance of a small number of genera of interest.

## 5 Conclusions

We demonstrated the significant impact of many different factors on the porcine microbiota across a gradient of authentic lifestyles. Beyond age category and diet, husbandry variables were equally shown to exert significant impacts on the microbiota. Among different systems, we also saw the dynamic and often compensatory roles of different factors in producing similar taxonomic effects. Key genera were observed to be associated with either conventional or rustic farming systems. Besides taxonomy, we observed the rustic-associated microbiota to be associated with decreased faecal water content, indicating increased gut transit time. The conventional-associated microbiota on the other hand, was associated with increased faecal water content, and may be better adapted to rapid, efficient fibre fermentation, as opposed to more complex, varied fermentation by a rustic-associated microbiota.

## Ethics approval

The experimental setup was approved by the KU Leuven Ethical Committee for Animal Experimentation (M024–2023).

## Availability of data and materials

All raw sequencing data are available as FASTQ files via the NCBI platform (accession number: PRJNA1308291). An interactive version of the enterotype profiles and their coding can also be accessed via marimo (https://marimo.io/p/@peiyang-huo/interactive-heatmap-for-latent-dirichlet-allocation). Although not novel, for the sake of transparency, coding for the analysis in R, and the input objects is available via the GitHub repository (https://github.com/lwcomer/microbioswine-WP1).

## Funding

Financial support for this project was provided by a KU Leuven grant (STG/21/045).

## CRediT authorship contribution statement

**Luke Comer:** Conceptualization, Data curation, Software, Visualization, Formal analysis, Investigation, Writing – original draft. **Peiyang Huo:** Software, Formal analysis, Visualization. **Camillo Colleluori:** Software, Formal analysis, Investigation. **Haoran Zhao:** Investigation. **Muhammad Zeeshan Akram:** Investigation. **Romario Florent Kpossou:** Investigation. **Ester Arévalo Sureda:** Investigation. **Jan Aerts:** Supervision. **Nadia Everaert:** Conceptualization, Supervision, Writing – review & editing.

## Declaration of competing interest

The authors declare that they have no known competing financial interests or personal relationships that could have appeared to influence the work reported in this paper.

## Appendix Supplementary materials

Image, application 1

Image, application 2

Image, application 3

Image, image 4 dummy alt text

Image, image 5 dummy alt text

Image, image 6 dummy alt text

Image, image 7 dummy alt text

Image, image 8 dummy alt text

Image, image 9 dummy alt text

Image, image 10 dummy alt text

Image, image 11 dummy alt text

Image, image 12 dummy alt text

Image, image 13 dummy alt text

Image, image 14 dummy alt text

Image, image 15 dummy alt text

Image, image 16 dummy alt text

Figure S1. Distribution of Faecal Water Content and the Classification of Porcine Stool Scores. Categorical porcine stool scores were given based on deviation from the median (porcine stool type 4). Type 1 represented faeces with the least water content, and type 6 samples with the highest water content. Stool score 1 was < 59.22% water content, score 2: 59.22–66.63%, score 3: 66.64–70.33%, score 4: 70.34–77.73%, score 5: 77.74–81.43%, and score 6: 81.43–88.83%. Image, image 17 dummy alt text
