# Supplementary material for: From forest to farm: the impact of a broad spectrum of lifestyles on the porcine gut microbiota
Source: Curr Res Microb Sci. 2026 Feb 28;10:100576. doi: 10.1016/j.crmicr.2026.100576 (PMC12969317; doi:10.1016/j.crmicr.2026.100576)
Supplement: Supplementary file 1 [file mmc1.docx]

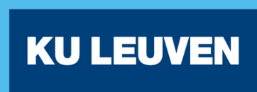

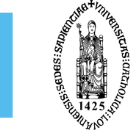
**SPECIMEN COLLECTION QUESTIONNAIRE**

Bag nr.

|  |  |  |  |  |  |
| --- | --- | --- | --- | --- | --- |

Subject ID:

|  |  |  |  |  |  |  |  |  |  |  |  |
| --- | --- | --- | --- | --- | --- | --- | --- | --- | --- | --- | --- |

Sanitel (national) ID:

Date ….../..…./……..…

Location Address:

…………………………………………………..

…………………………………………………..

…………………………………………………..

|  |  |  |  |
| --- | --- | --- | --- |

Location ID:

ID of specimen/s:

|  |  |  |  |  |  |  |  |  |  |  |  |
| --- | --- | --- | --- | --- | --- | --- | --- | --- | --- | --- | --- |

|  |  |  |  |  |  |  |  |  |  |  |  |
| --- | --- | --- | --- | --- | --- | --- | --- | --- | --- | --- | --- |

**OVERVIEW**

Species………………………………………..

Breed…………………………………………..

Sex: Male / Female

Date of birth, age ….../..…./……..… *or* …………weeks/months/years

Country of birth…………………………………

Place (establishment) of birth:

Same as current residence? Yes / No

If no, establishment of birth………………………………………….

Date of arrival ….../..…./……..…

Phenotype description:

Photograph

Bodyweight …..……… (kg) (date: ….../..…./……..…)

**BREED OVERVIEW**

Breed line (if known)………………………………………………………

Country of origin…………………………………………………………...

Traditional use (lean/fatty, lard/bacon…)………………………………..

**ENVIRONMENT**

Establishment type (farm/smallholding/zoo?):

Farm Smallholding/personal Zoo Other…………………..

Intensity of husbandry? Small-scale / Intensive

Primary living? Inside / Outside

If inside: additional outside access? Yes / No

If outside: environment type (i.e. field, woods…) …………………………..

Access to soil? Yes / No

Bedding/floor material…………………………………………

Slatted floor present? Yes / No

Number of animals in the same enclosed space/pen/barn…………………………..

Housed with other species of animal? Yes / No

If yes, what?..................................................

**WEANING HISTORY**

Age at weaning………………………days

Was this animal ever given formula milk? Yes / No

Was creep feed given at weaning? Yes / No

If yes, what?...................................................

Creep feed consistency? Solid / Liquid

**DIET**

Daily feed regimen:

Feed brand/name……………………………………………………………….

Ingredients:

Feed type? Organic / Standard

Additional access to environmental food? Yes / No

If yes, what?................................................

Additional dietary supplementation? Yes / No

If yes, what? ………………………………….

**MEDICAL HISTORY**

Known medical conditions? Yes / No

If yes, what? ………………………………….

Past medication and why:

Past antibiotic use (in the past 6 months, or earlier if known) and why:

Worming/anti-parasite treatment in the last 6 months? Yes / No

Immunisation history (if known):

If female: has she farrowed previously? Yes / No

If yes, how long ago? ……………weeks/months/years

Is she gestating now? Yes / No

Is she nursing now? Yes / No

Parity…………………..

If male: castrated? Yes / No

If yes, type? Chemical / Physical

**SUBJECT ID CODES OF RELATIVES/COHABITANTS**

| **Subject ID** | | | | | | **Relationship to current subject** | |
| --- | --- | --- | --- | --- | --- | --- | --- |
| - | - | - | - |  |  |  |  |
| - | - | - | - |  |  |  |  |
| - | - | - | - |  |  |  |  |
| - | - | - | - |  |  |  |  |
| - | - | - | - |  |  |  |  |
| - | - | - | - |  |  |  |  |
| - | - | - | - |  |  |  |  |
| - | - | - | - |  |  |  |  |
| - | - | - | - |  |  |  |  |

**OTHER INFORMATION OF NOTE (IF APPLICABLE)**
