# Supplementary material for: From forest to farm: the impact of a broad spectrum of lifestyles on the porcine gut microbiota
Source: Curr Res Microb Sci. 2026 Feb 28;10:100576. doi: 10.1016/j.crmicr.2026.100576 (PMC12969317; doi:10.1016/j.crmicr.2026.100576)
Supplement: Supplementary file 3 [file mmc3.docx]

**Supplementary Tables**

| **Age category** | **Chronological age** |
| --- | --- |
| Lactation (suckling) | Birth – weaning |
| Nursery | Weaning – 10 weeks |
| Growing | 10 – 18 weeks |
| Finishing | 18 – 26 weeks |
| Mature | > 26 weeks |

**Table S1. Standardised Age Categories for the Classification of Each Animal in the Present Study**

| **Category** | **Crude fibre (%)** | **Crude protein (%)** |
| --- | --- | --- |
| Very high | > 8 | NA |
| High | 4 – 8 | > 20 |
| Medium | 2 – 4 | 15 – 20 |
| Low | < 2 | < 15 |

**Table S2. Standardised Dietary Categories for the Classification of Each Animal According to Dietary Crude Fibre and Protein.**

| **Component** | **Final concentration in buffer** |
| --- | --- |
| CaCl_2_·2H_2_O | 15.88 mg/L |
| MnCl·4H_2_O | 12.00 mg/L |
| CoCl_2_·6H_2_O | 1.20 mg/L |
| FeCl_3_·6H_2_O | 0.96 mg/L |
| NaHCO_3_ | 8.29 g/L |
| NH_4_Cl | 637 mg/L |
| Na_2_HPO_4_ | 1.36 g/L |
| KH_2_PO_4_ | 1.47 g/L |
| MgSO_4_·7H_2_O | 142.50 mg/L |
| Resazurin (C_12_H_7_NO_4_) | 1.00 mg/L |

**Table S3. Composition of Modified Fermentation Buffer Used in the Present Study**

| **Core Genera** | **Core Species** |
| --- | --- |
| *Prevotella, Clostridium, Lactobacillus,* unclassified Lachnospiraceae, unclassified Oscillospiraceae, *Terrisporobacter, Cryptobacteroides, Treponema, Limosilactobacillus, CAG-83, Roseburia, Romboutsia,* unclassified UBA932, unclassified Muribaculaceae, *Blautia, GCA-900199385, Ruminococcus,* unclassified Ruminococcaceae, *Alloprevotella,* unclassified Bacteroidaceae, *Turicibacter, Phascolarctobacterium,* unclassified Actualibacteraceae, unclassified Oscillospirales, *Bariatricus, Eubacterium, Dysosmobacter,* unclassified Anaerovoracaceae*, Faecalicoccus,* unclassified Clostridia, *Fimenecus,* unclassified CAG-508, *RUG13077, Desulfovibrio, Copromorpha* | *Bariatricus comes, CAG-83 sp001916855, Clostridium sp001916075, Cryptobacteroides sp002438635, Eubacterium coprostanoligenes, Faecalicoccus acidiformans, GCA-900199385 sp900322155, Phascolarctobacterium succinatutens, Prevotella sp002251245, RUG13077 sp902785485, Romboutsia timonensis, Terrisporobacter sp902363255, Turicibacter sanguinis* |

**Table S4. Genera and Species Belonging to the Core Suid Microbiota**

Taxa belonging to the core microbiota were those defined as present in > 95% individuals.

|  | **A** | | **B** | | **C** | | **R_MAX_** | | | **T_MAX_** | |
| --- | --- | --- | --- | --- | --- | --- | --- | --- | --- | --- | --- |
| **01BE** | 321.63^ab^ | 13.15 | 13.48^ab^ | 0.44 | 2.74^a^ | 0.11 | 18.7^ab^ | 0.81 | | 10.18^a^ | 0.56 |
| **12NL** | 365.56^a^ | 11.45 | 11.00^bc^ | 0.53 | 2.54^a^ | 0.11 | 24.8^ac^ | 0.42 | | 7.89^ab^ | 0.49 |
| **23BE** | 338.21^ab^ | 6.68 | 7.50^d^ | 0.20 | 2.58^a^ | 0.27 | 34.3^d^ | 2.31 | | 5.26^b^ | 0.45 |
| **24BE** | 342.81^ab^ | 13.71 | 8.44^cd^ | 0.16 | 3.14^a^ | 0.16 | 35.3^d^ | 1.03 | | 6.81^b^ | 0.25 |
| **30NL** | 320.86^ab^ | 22.42 | 10.37^acd^ | 0.80 | 3.09^a^ | 0.08 | 26.7^c^ | 1.19 | | 8.35^ab^ | 0.72 |
| **Wild** | 279.10^b^ | 2.49 | 14.29^b^ | 0.92 | 1.19^b^ | 0.07 | 14.1^b^ | 1.16 | | 1.77^c^ | 0.69 |
|  |  |  |  |  |  |  |  |  | |  |  |
| ***P*** | 0.045 | | < 0.001 | | < 0.001 | | < 0.001 | | < 0.001 | | |

**Table S5. Kinetic Gas Production Parameters for the Galactooligosaccharide Fermentation.**

Kinetic parameters are included, alongside the standard error of the mean per group. ANOVA was performed to determine significant differences per group, with pairwise comparisons also performed; significant differences between groups are denoted with differing subscripts. A: the maximum gas volume (mL/g), B: the inflection parameter, C: the rate constant of gas production (h), R_MAX_: the maximum rate of gas production (mL/h/g), T_MAX_: the time taken to reach the maximum rate of gas production (h
