# Supplementary figures and images for: From forest to farm: the impact of a broad spectrum of lifestyles on the porcine gut microbiota
Source: Curr Res Microb Sci. 2026 Feb 28;10:100576. doi: 10.1016/j.crmicr.2026.100576 (PMC12969317; doi:10.1016/j.crmicr.2026.100576)

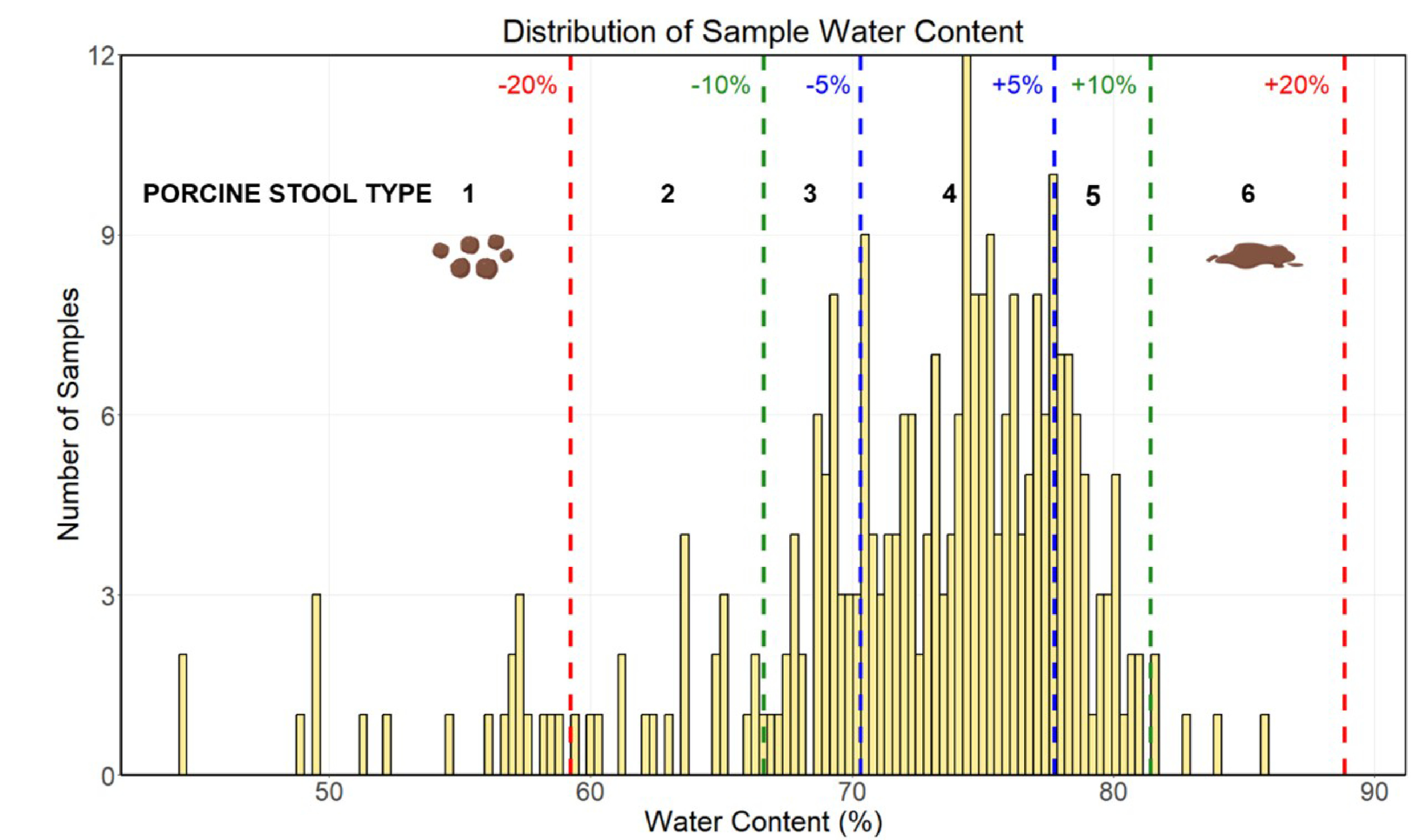

Supplement: Supplementary file 4 [file mmc4.jpg]

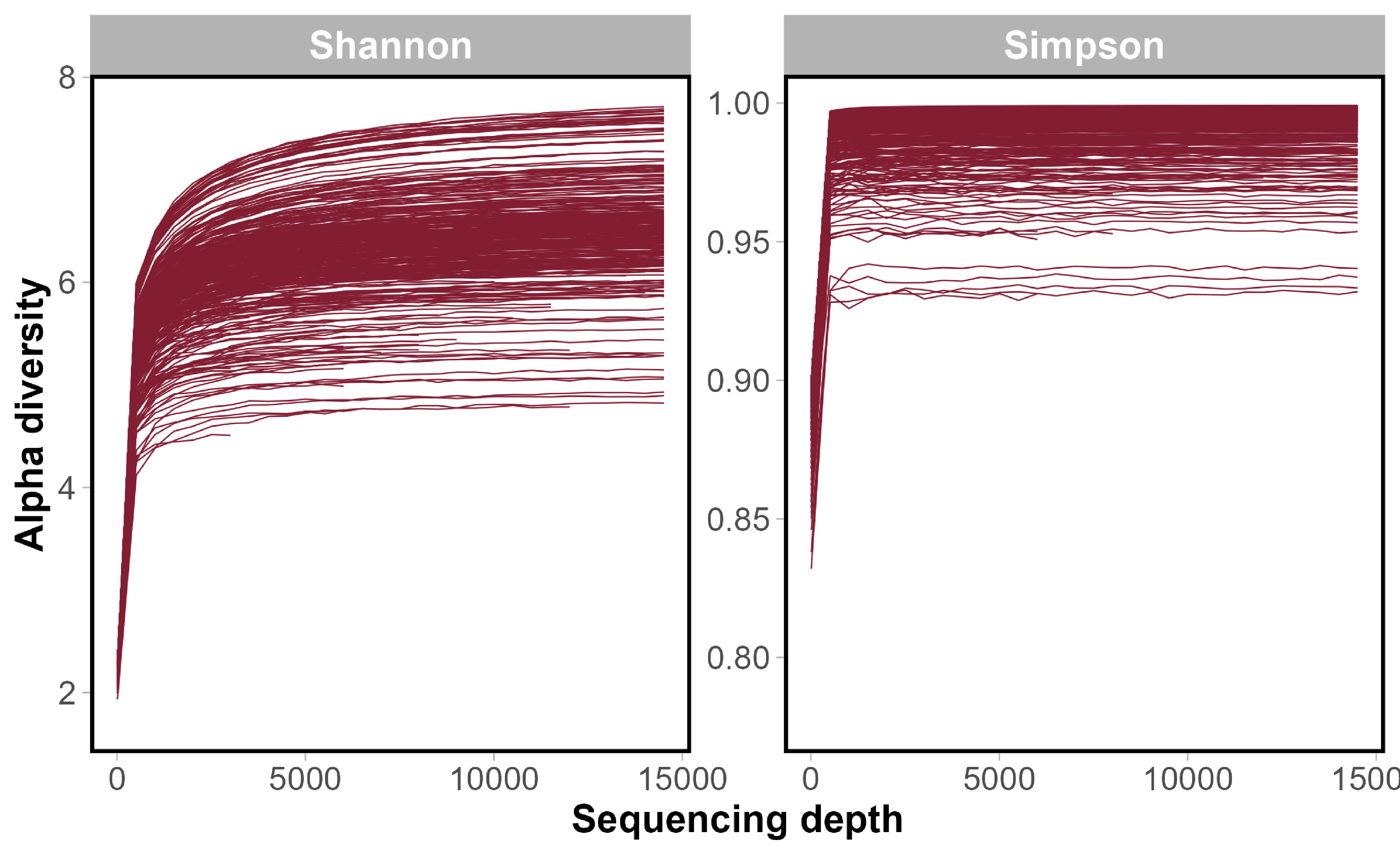

Supplement: Supplementary file 5 [file mmc5.jpg]

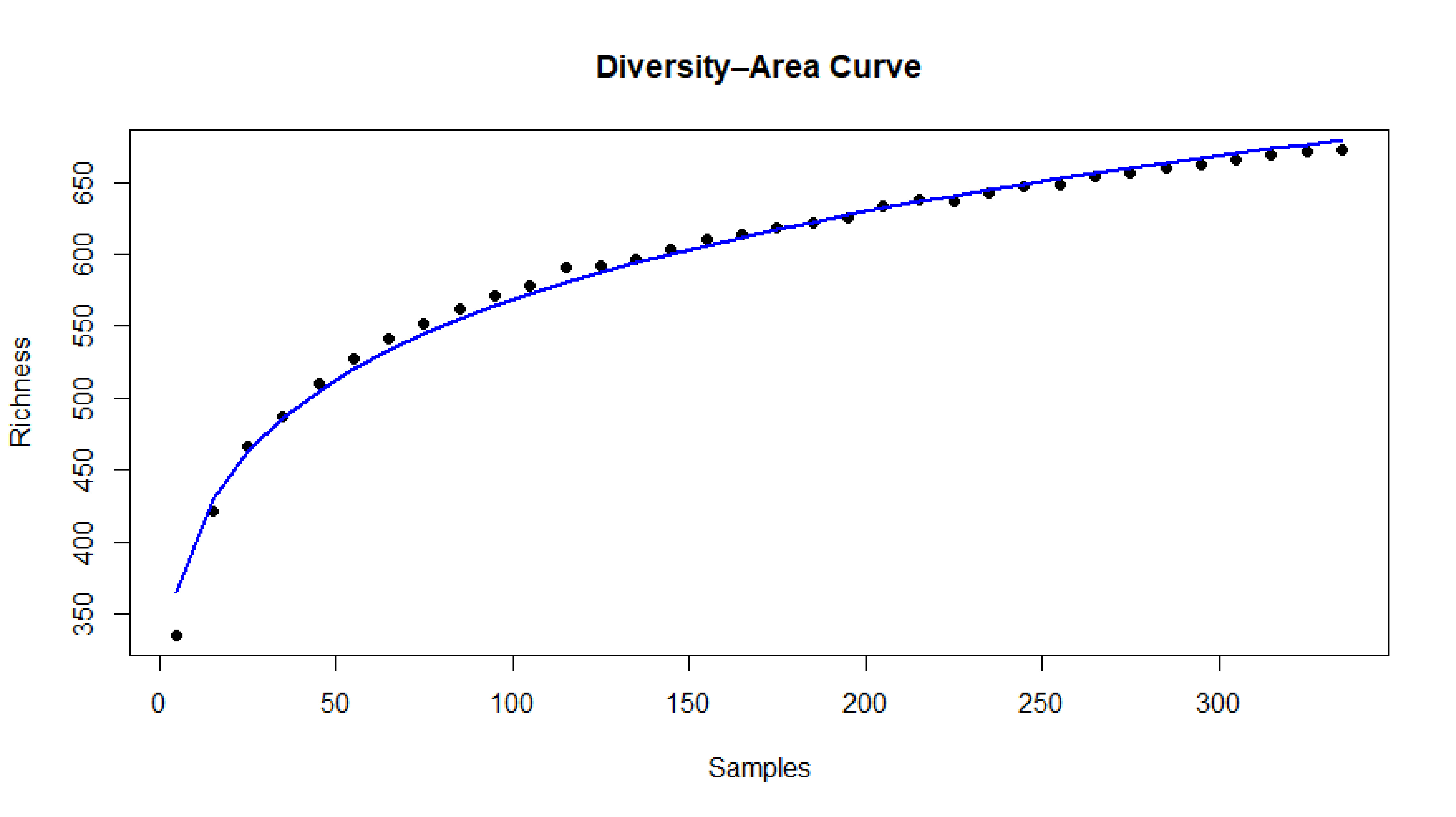

Supplement: Supplementary file 6 [file mmc6.jpg]

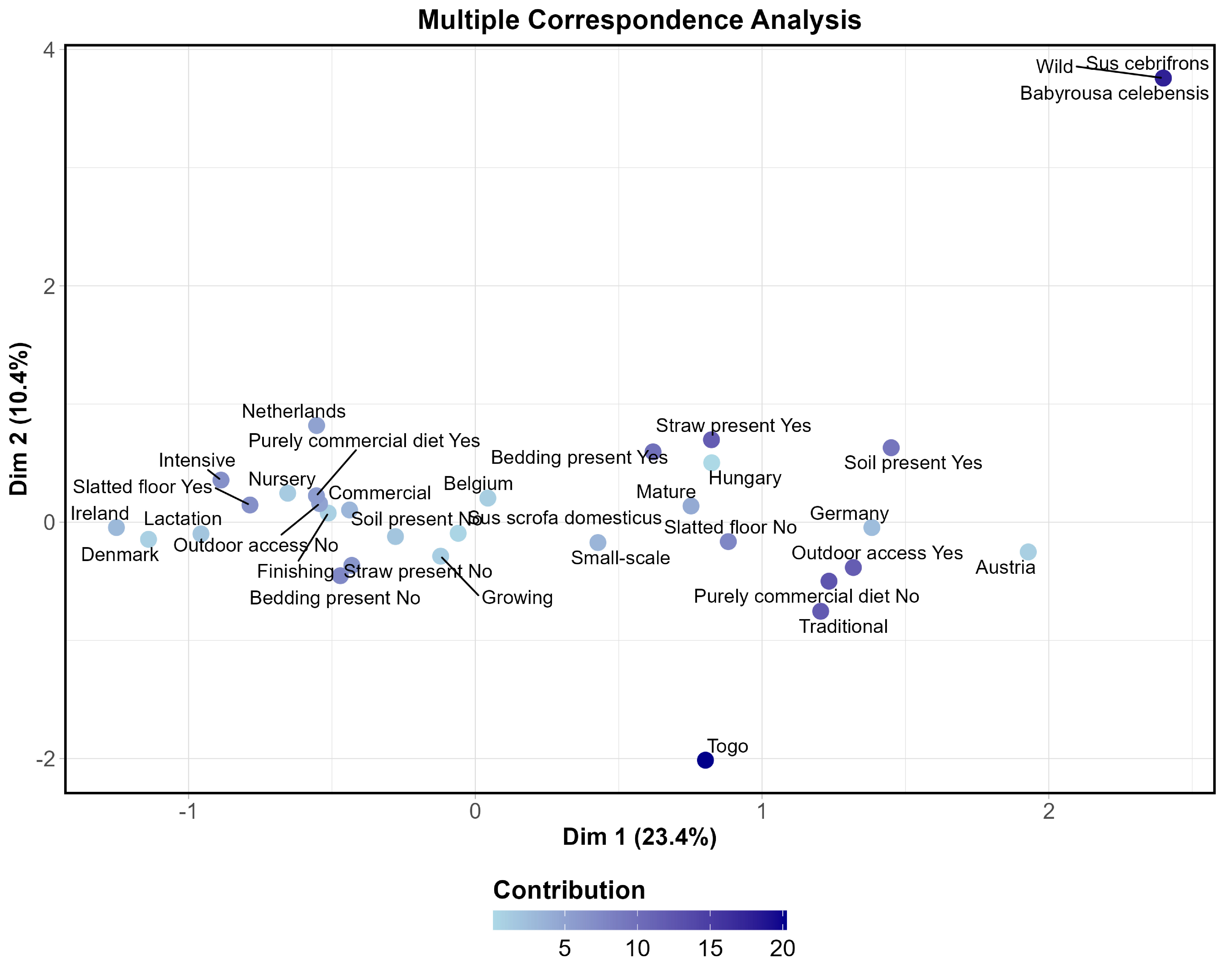

Supplement: Supplementary file 7 [file mmc7.jpg]

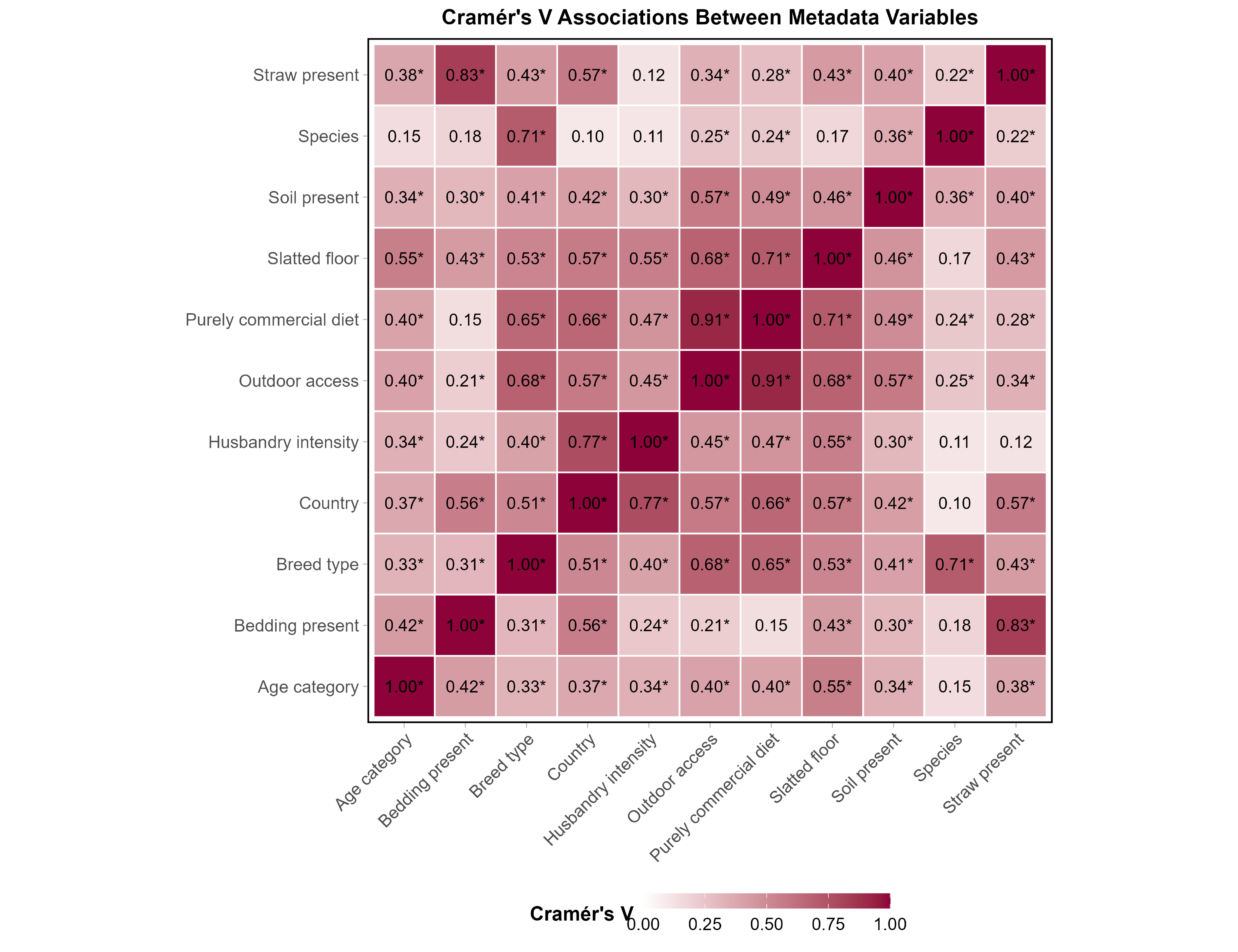

Supplement: Supplementary file 8 [file mmc8.jpg]

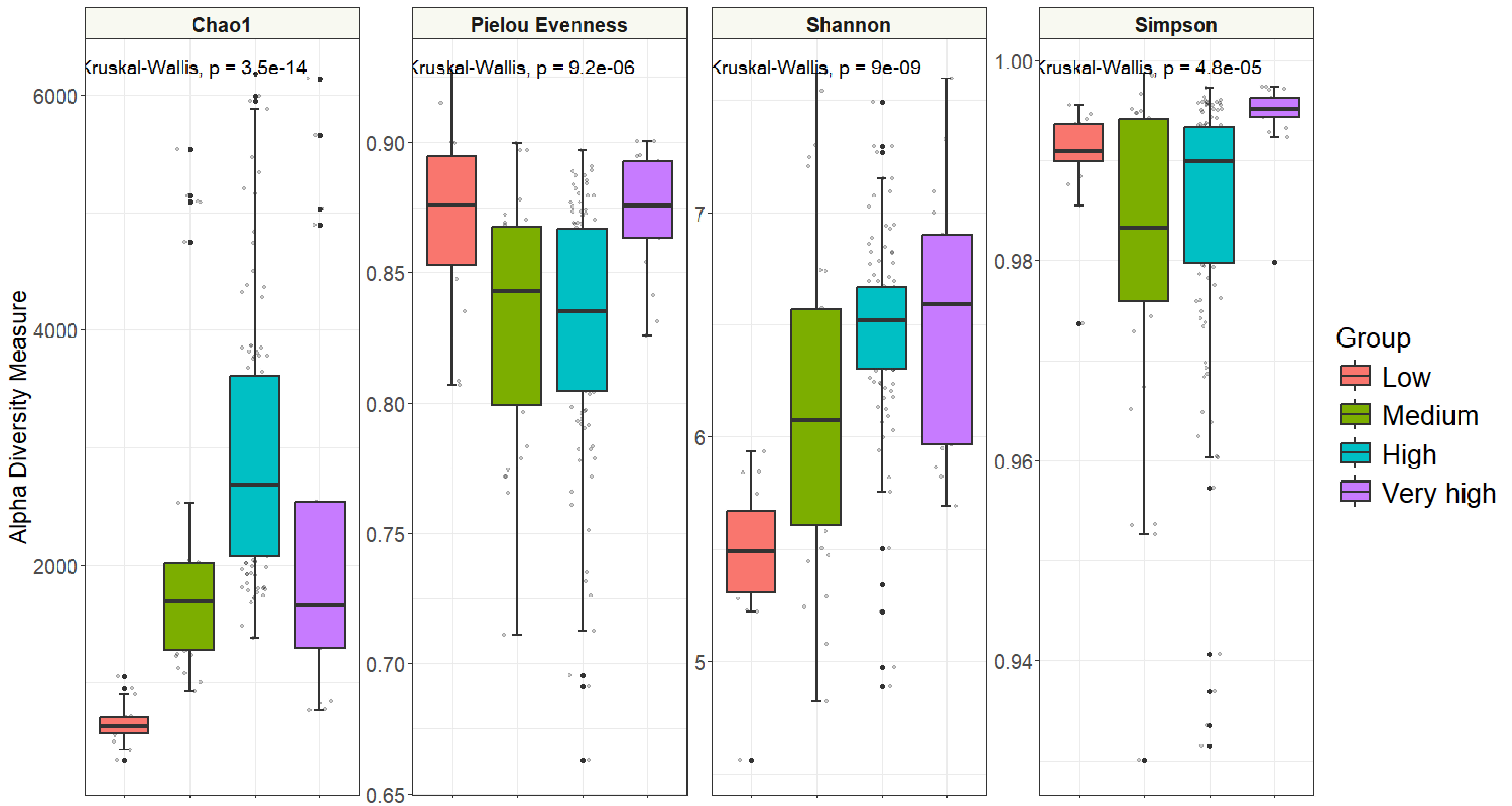

Supplement: Supplementary file 9 [file mmc9.jpg]

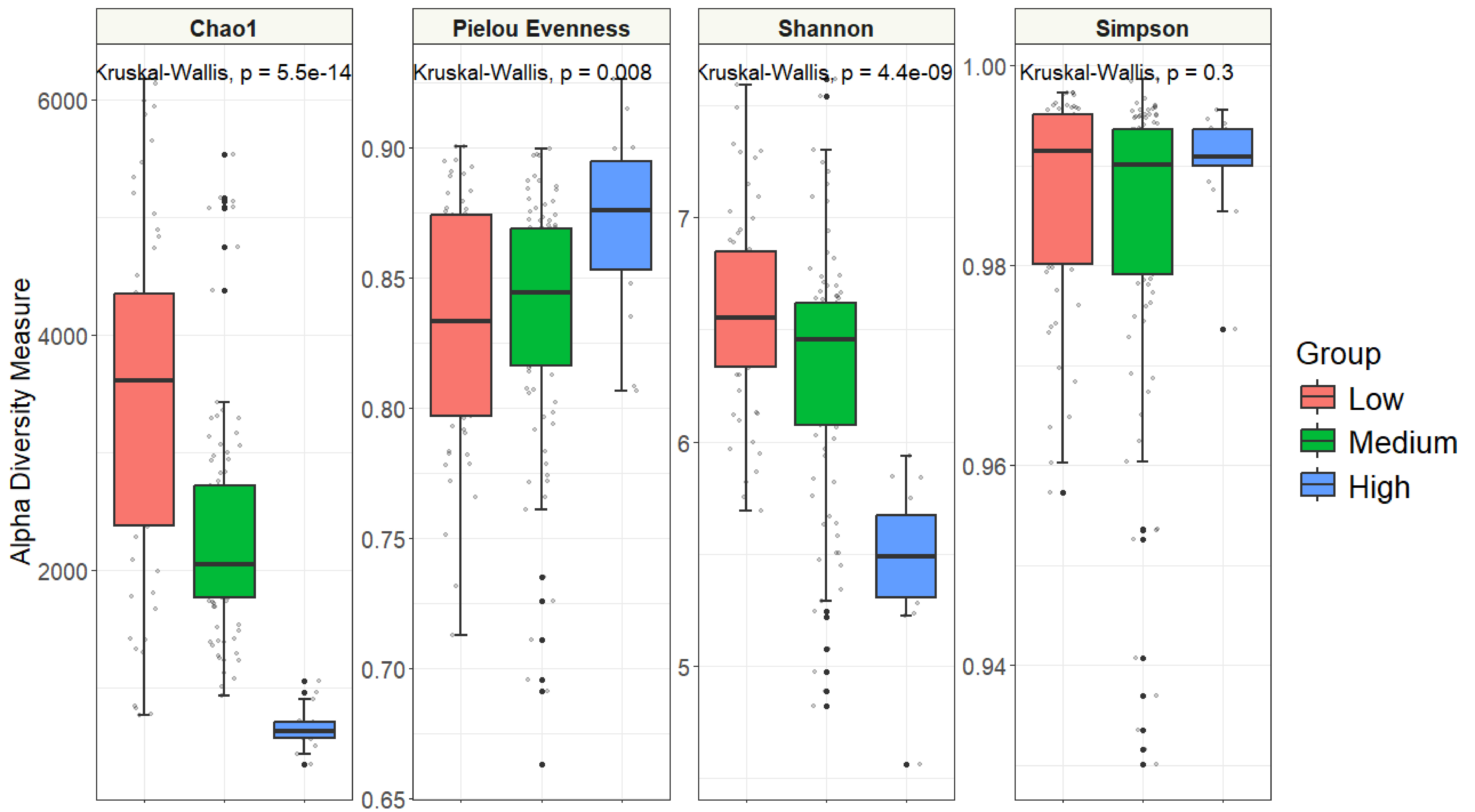

Supplement: Supplementary file 10 [file mmc10.jpg]

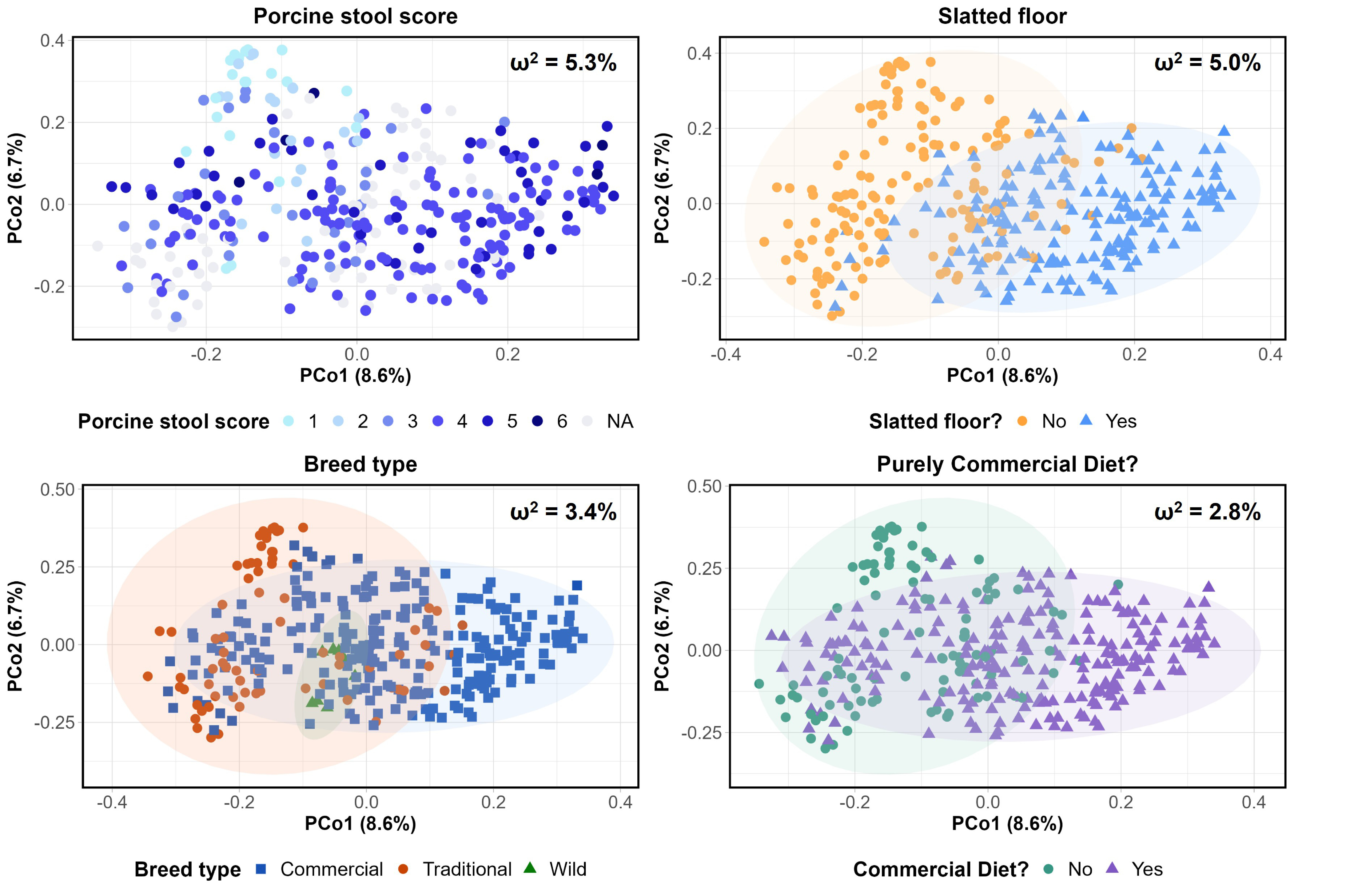

Supplement: Supplementary file 11 [file mmc11.jpg]

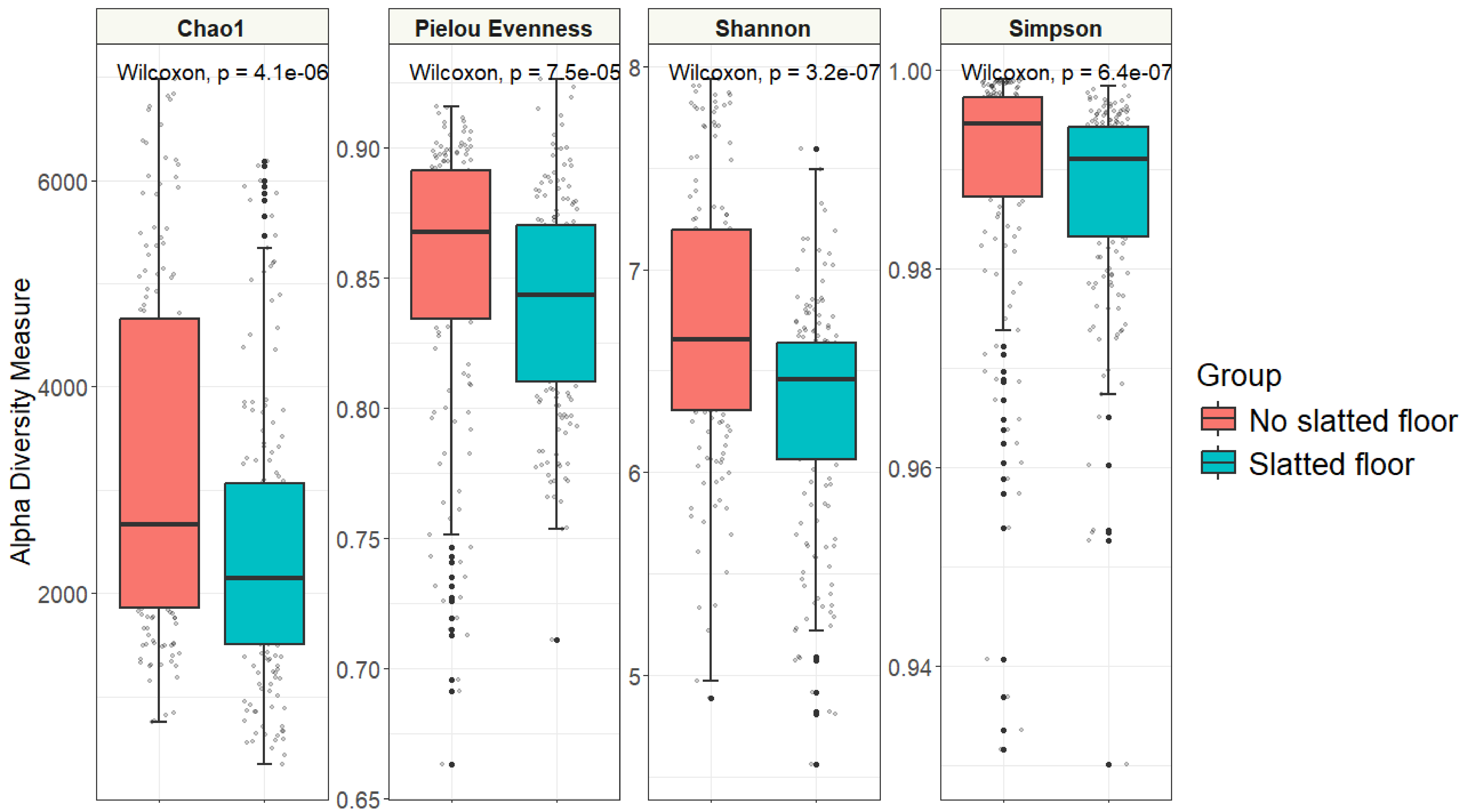

Supplement: Supplementary file 12 [file mmc12.jpg]

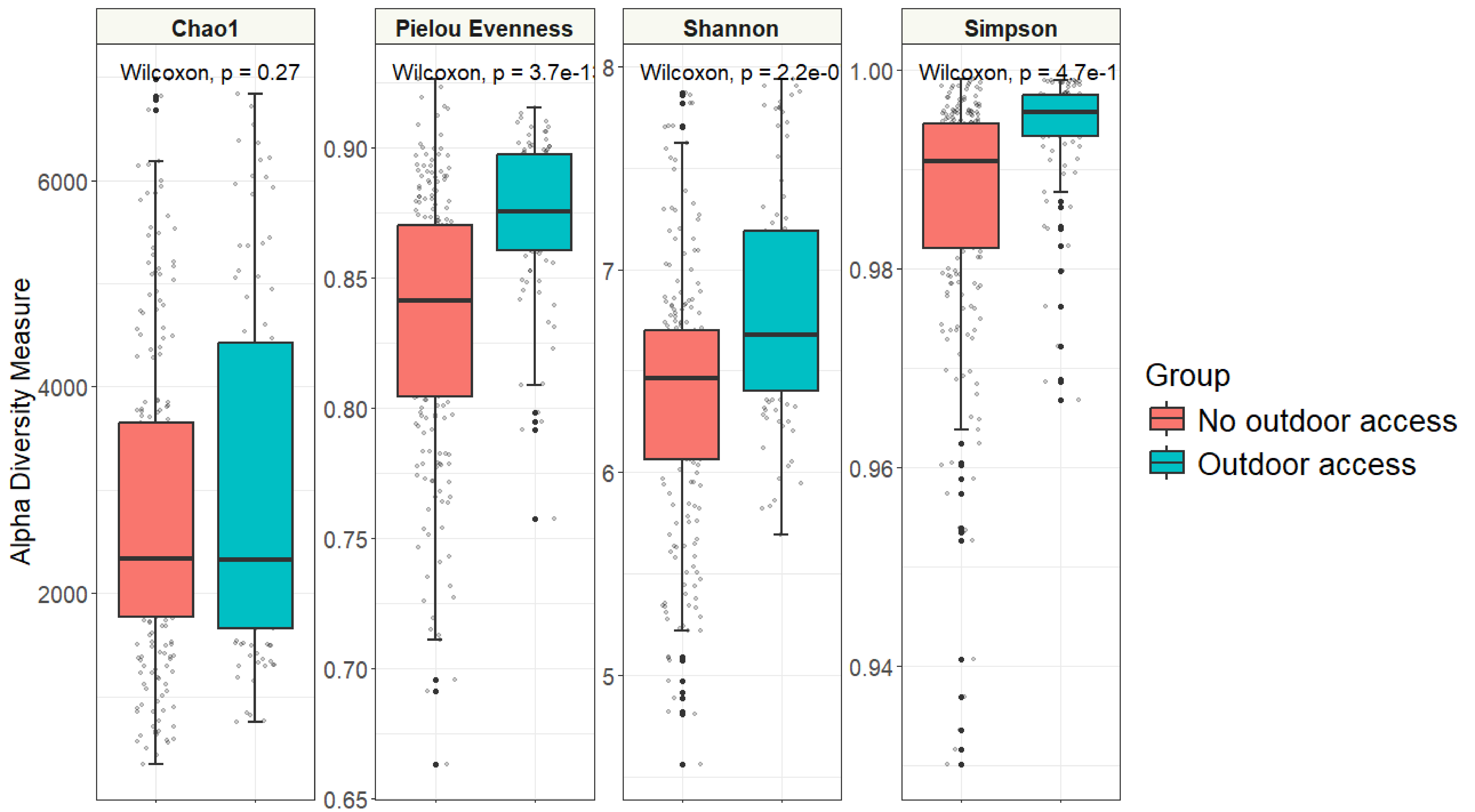

Supplement: Supplementary file 13 [file mmc13.jpg]

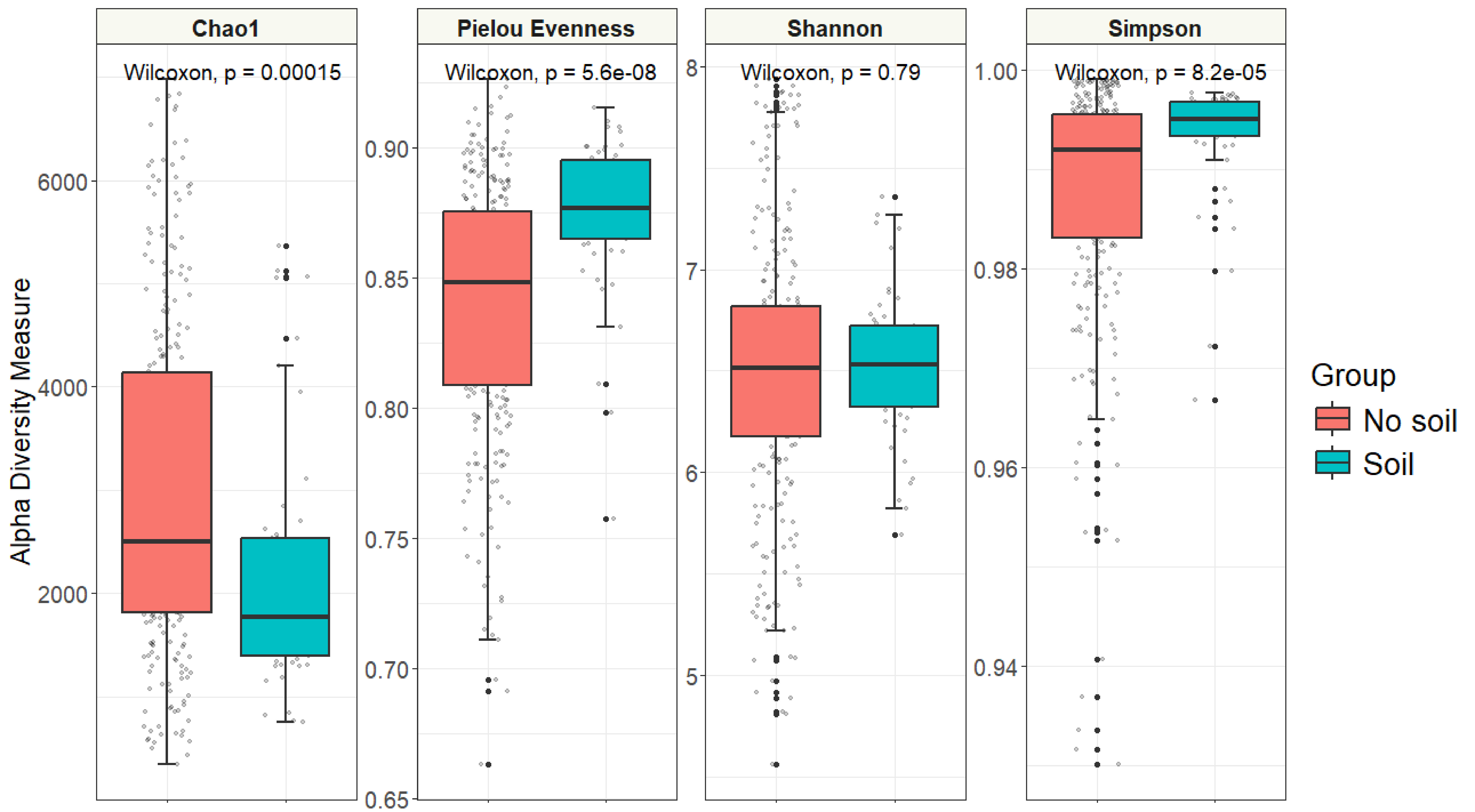

Supplement: Supplementary file 14 [file mmc14.jpg]

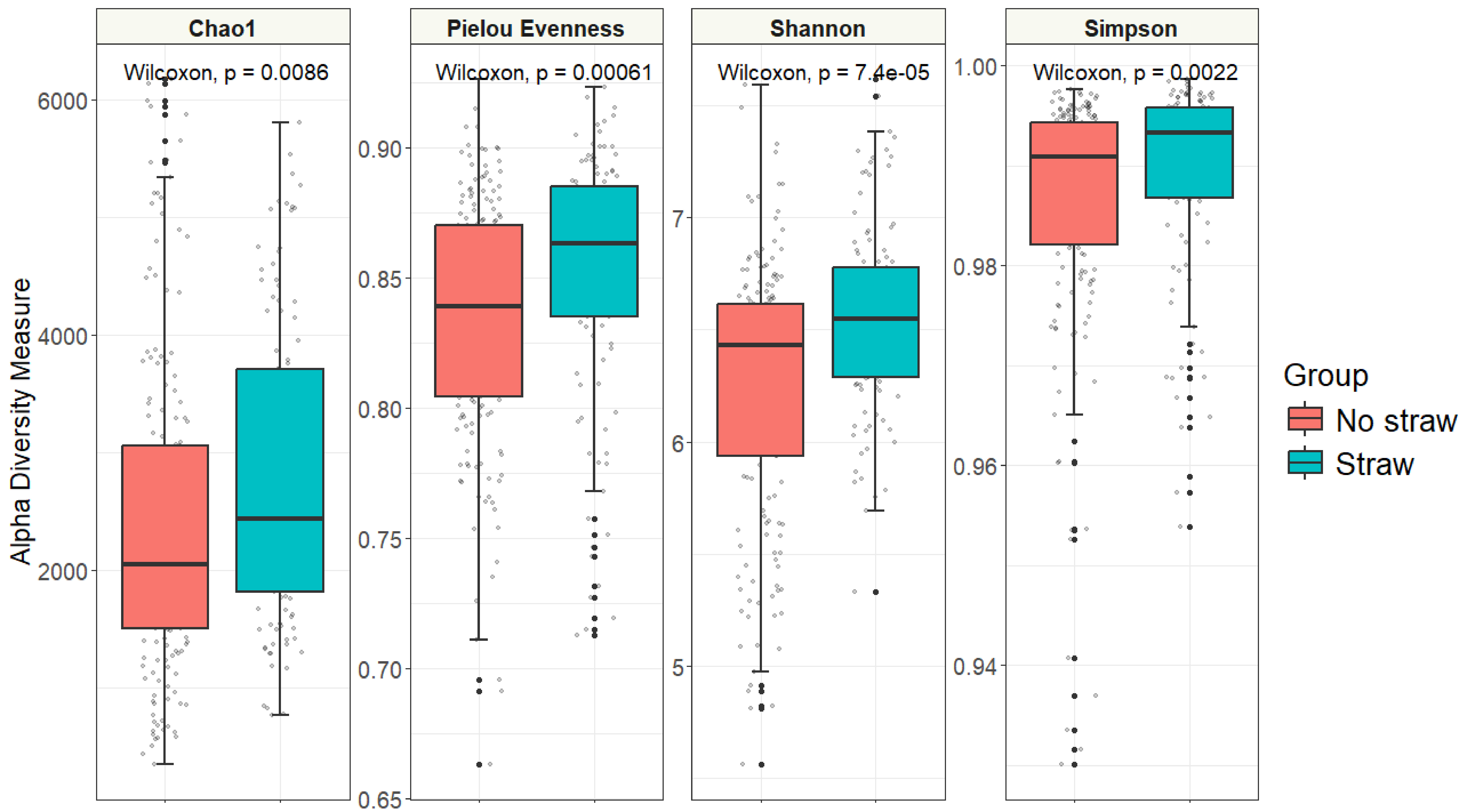

Supplement: Supplementary file 15 [file mmc15.jpg]

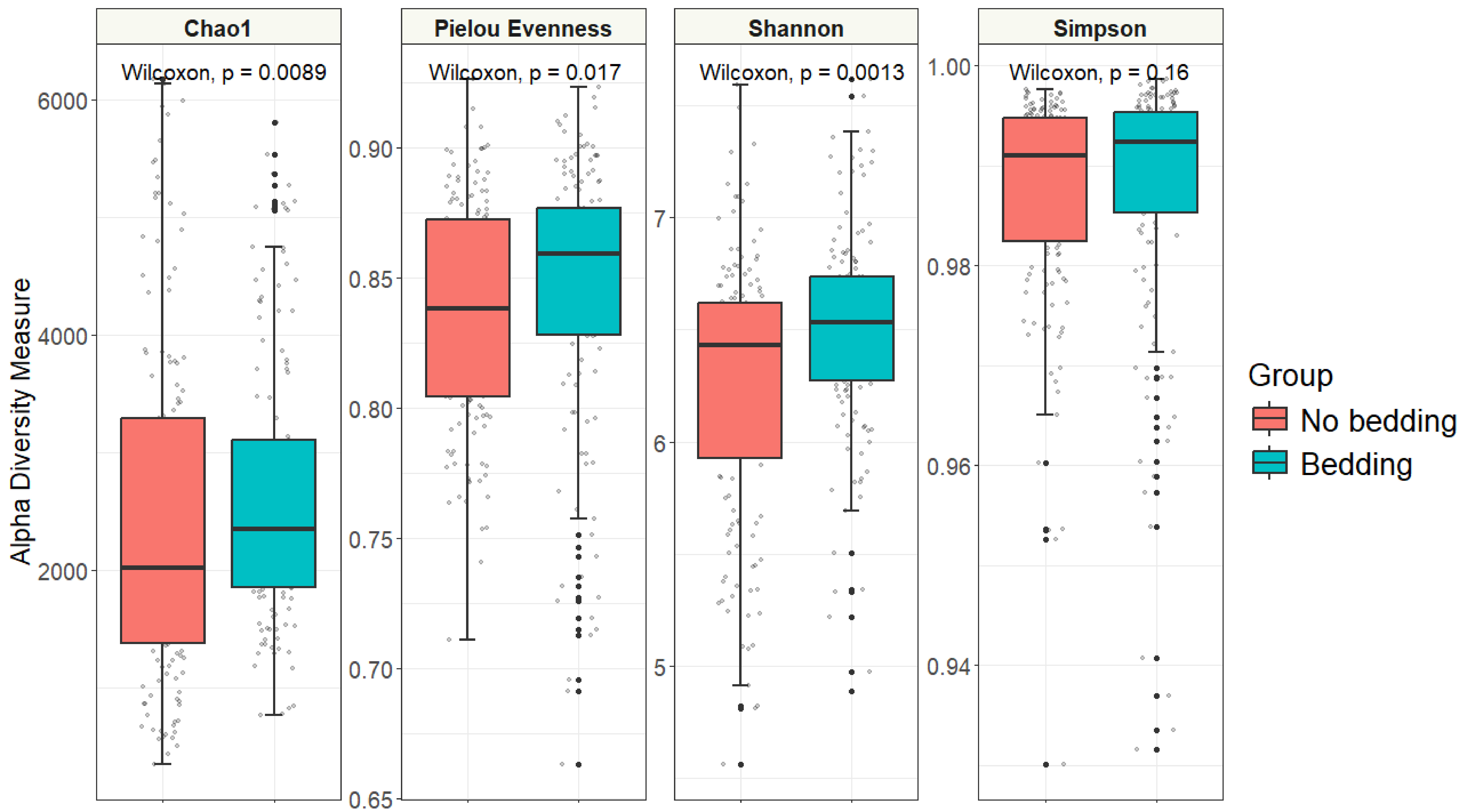

Supplement: Supplementary file 16 [file mmc16.jpg]

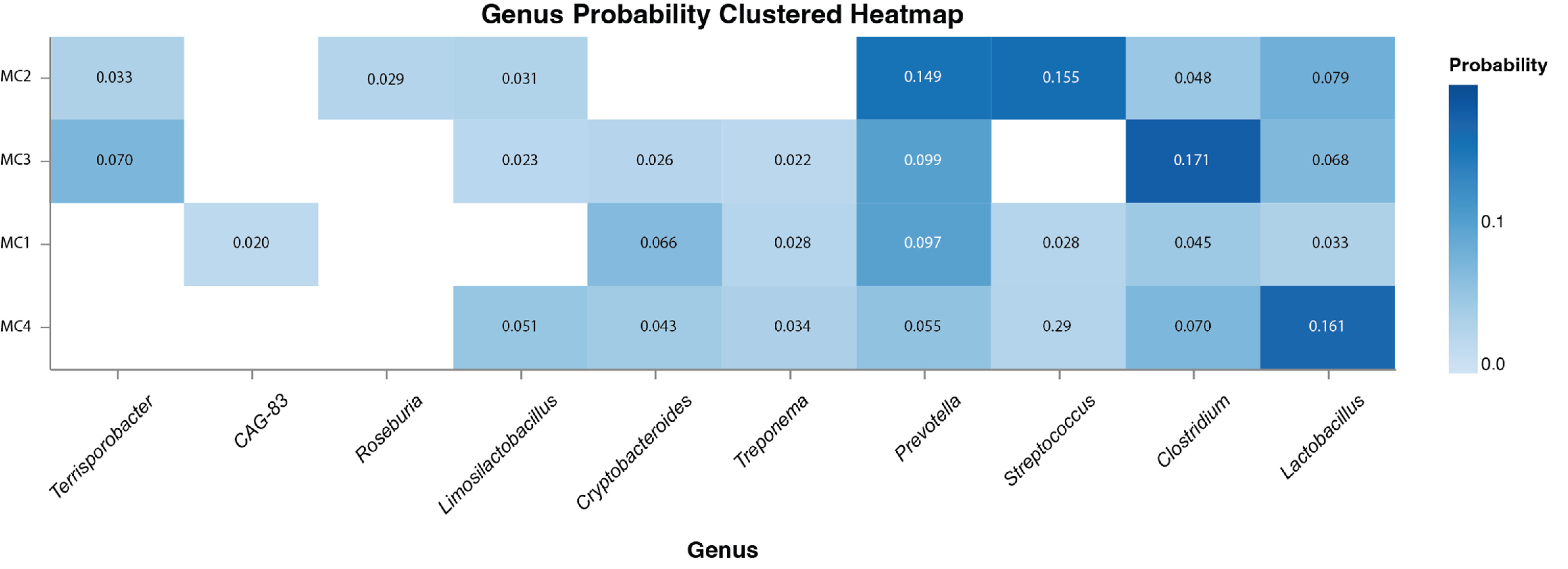

Supplement: Supplementary file 17 — Figure S1. Distribution of Faecal Water Content and the Classification of Porcine Stool Scores. Categorical porcine stool scores were given based on deviation from the median (porcine stool type 4). Type 1 represented faeces with the least water content, and type 6 samples with the highest water content. Stool score 1 was < 59.22% water content, score 2: 59.22–66.63%, score 3: 66.64–70.33%, score 4: 70.34–77.73%, score 5: 77.74–81.43%, and score 6: 81.43–88.83%. [file mmc17.jpg]
